# The Mechanism of Exogenous Salicylic Acid and 6-Benzylaminopurine Regulating the Elongation of Maize Mesocotyl

**DOI:** 10.3390/ijms25116150

**Published:** 2024-06-03

**Authors:** Xue Qi, Zelong Zhuang, Xiangzhuo Ji, Jianwen Bian, Yunling Peng

**Affiliations:** 1 College of Agronomy, Gansu Agricultural University, Lanzhou 730070, China; 2 Gansu Provincial Key Laboratory of Aridland Crop Science, Gansu Agricultural University, Lanzhou 730070, China; 3 Gansu Key Laboratory of Crop Improvement & Germplasm Enhancement, Gansu Agricultural University, Lanzhou 730070, China

**Keywords:** mesocotyl, deep-sowing, maize (*Zea mays* L.), SA, 6-BA

## Abstract

The elongation of the mesocotyl plays an important role in the emergence of maize deep-sowing seeds. This study was designed to explore the function of exogenous salicylic acid (SA) and 6-benzylaminopurine (6-BA) in the growth of the maize mesocotyl and to examine its regulatory network. The results showed that the addition of 0.25 mmol/L exogenous SA promoted the elongation of maize mesocotyls under both 3 cm and 15 cm deep-sowing conditions. Conversely, the addition of 10 mg/L exogenous 6-BA inhibited the elongation of maize mesocotyls. Interestingly, the combined treatment of exogenous SA–6-BA also inhibited the elongation of maize mesocotyls. The longitudinal elongation of mesocotyl cells was the main reason affecting the elongation of maize mesocotyls. Transcriptome analysis showed that exogenous SA and 6-BA may interact in the hormone signaling regulatory network of mesocotyl elongation. The differential expression of genes related to auxin (IAA), jasmonic acid (JA), brassinosteroid (BR), cytokinin (CTK) and SA signaling pathways may be related to the regulation of exogenous SA and 6-BA on the growth of mesocotyls. In addition, five candidate genes that may regulate the length of mesocotyls were screened by Weighted Gene Co-Expression Network Analysis (WGCNA). These genes may be involved in the growth of maize mesocotyls through auxin-activated signaling pathways, transmembrane transport, methylation and redox processes. The results enhance our understanding of the plant hormone regulation of mesocotyl growth, which will help to further explore and identify the key genes affecting mesocotyl growth in plant hormone signaling regulatory networks.

## 1. Introduction

Maize (*Zea mays* L.) is a crucial crop for food and feed in China, playing a significant role in ensuring national food security and agricultural production. Currently, crop growth is affected by various abiotic stresses, including drought, salinity and low temperature. Among these, drought is the most significant factor that impacts the growth and yield of maize [1]. Drought can cause a 20~30% reduction in maize yield every year and even 30~35% in severe drought years [2]. Therefore, taking effective measures for drought prevention and relief to improve the crop’s ability to withstand drought is a crucial approach to ensuring consistent and high corn yields.

Appropriately increasing the depth of sowing is one of the effective measures for preventing drought and preserving soil moisture during the maize seedling stage [3]. Mesocotyl elongation is a critical factor in facilitating the emergence of maize seeds under deep-sowing conditions [4]. Studies indicate that mesocotyl length is influenced by both genetic background and the external environment, with a complex genetic mechanism at play [5]. Some scholars suggest that light is an important environmental factor in the regulation of plant growth and development, and the length of the mesocotyl is related to light stimulation [6]. Light treatment can increase the activity of phytochrome-mediated PAO (polyamine oxidase) in maize mesocotyls, which in turn causes an increase in H_2_O_2_ content, resulting in a decrease in cell wall ductility and inhibition of mesocotyl elongation [7]. However, dark conditions are conducive to the elongation of mesocotyls. Compared with light conditions (far red, red), the cell elongation ability of mesocotyls under dark conditions is the strongest [8]. At the same time, plant hormones have a certain regulatory effect on mesocotyl length. Under conditions of deep sowing, the application of exogenous auxin (IAA) can significantly promote the elongation of mesocotyls and increase the content of endogenous IAA [9]. IAA is involved in the elongation of maize mesocotyl cells induced by light and auxin by promoting the expression of ABP1 (auxin binding protein 1) gene [10]. Similarly, exogenous gibberellin (GA_3_) has a positive regulatory effect on the mesocotyl length and coleoptile length of deep-sowing maize inbred lines [11]. GA receptor GIDI (GA-insensitive dwarf1), MYB (v-myb avian myeloblastosis viral oncogene homolog) transcription factor and gene encoding DELLA (aspartic acid–glutamic acid–leucine–leucine–alanine) protein DWRF8 play a key role in GA_3_-induced mesocotyl elongation under deep-sowing conditions. GA may also promote mesocotyl elongation by inhibiting the abscisic acid (ABA) signal response pathway [12]. In addition, exogenous brassinosteroid (BR) and IAA have a synergistic effect and promote mesocotyl elongation by acidifying and relaxing the epidermal cell wall [13]. Exogenous ABA [14], cytokinin (CTK) [15], ethylene (ETH) [16], jasmonic acid (JA) [17] and SA signal-related transcription factors [18] also play a regulatory role in mesocotyl length. However, the hormone network regulating the length of maize mesocotyls is not well understood.

Salicylic acid (SA) is a naturally occurring phenolic compound and a key signaling molecule that regulates various physiological and morphological responses in plants [19]. 6-benzylaminopurine (6-BA) is a synthetic cytokinin growth regulator, which plays an important regulatory role in plant growth and development and resistance to environmental stresses, such as drought, high temperature, low temperature, waterlogging and salt [20]. At present, the effects of exogenous SA and 6-BA on maize mesocotyls have not been reported. Therefore, we conducted cytological observation and transcriptome sequencing of mesocotyls treated with exogenous hormones under dark conditions using deep-seeding tolerant and deep-seeding sensitive inbred lines as the materials. The effects of exogenous SA and 6-BA on the growth of mesocotyl cells and the changes in differential gene expression levels were analyzed, and the physiological response and molecular regulation mechanism of exogenous SA and 6-BA on the growth of maize mesocotyls were discussed. It provides a reference for analyzing the regulatory network of exogenous hormones on mesocotyl elongation.

## 2. Results

### 2.1. Effects of Different Concentrations of Exogenous SA, 6-BA and SA–6-BA on Deep-Sowing Sensitive Maize Inbred Lines

**Figure 1:**
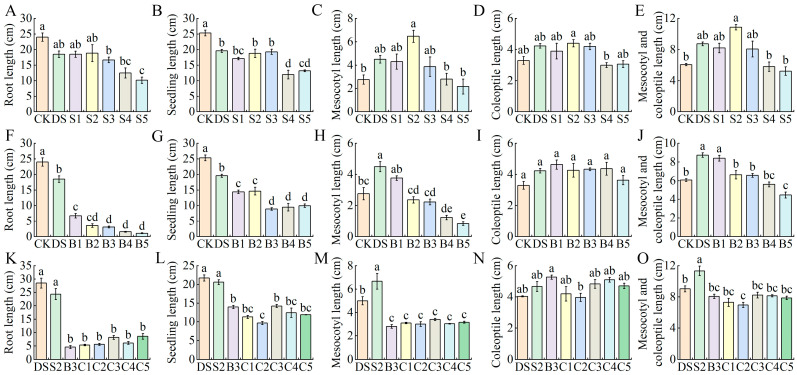
Changes in phenotypic traits of maize inbred lines Zi330 treated with different concentrations of exogenous hormones. (**A**–**E**) Phenotypic indicators under exogenous SA treatment. (**F**–**J**) Phenotypic indexes under exogenous 6-BA treatment. (**K**–**O**) Phenotypic indicators under exogenous SA–6-BA treatment. CK = 3 cm, DS = 15 cm. Different lowercase letters represent significant differences between treatments by Tukey’s test (*p* < 0.05).

Compared with the normal sowing depth, the mesocotyl length, coleoptile length and the sum of the mesocotyl and coleoptile lengths of Zi330 under deep-sowing conditions increased significantly, with an increase of 28.3%~62.7% (Figure 1C–E); the root length and seedling length decreased significantly by 22.9% and 22.7% (Figure 1A,B). The effects of different concentrations of exogenous SA on the growth parameters of maize seedlings were promoted at low concentrations (0.1~0.5 mmol/L) and inhibited at high concentrations (1~2 mmol/L) (Figure 1A–E). When 0.25 mmol/L SA (S2) was applied, the mesocotyl length and the sum of the mesocotyl length and coleoptile length of inbred line Zi330 changed the most, which were 1.44 and 1.24 times higher than that of DS, respectively. The optimal concentration of exogenous SA treatment was determined to be 0.25 mmol/L (S2) based on the average value of the membership function (Table S1). Different concentrations of exogenous 6-BA inhibited the growth of maize seedlings (Figure 1F–J). After the application of 6-BA, the root length, seedling length, mesocotyl length and the sum of the mesocotyl and coleoptile lengths of Zi330 were significantly reduced, and the reduction increased with concentration. However, the application of exogenous 6-BA had no significant effect on coleoptile length. The average value of the membership function of each index gradually decreased with the increase in concentration (Table S2). In order to facilitate the sampling and detection of mesocotyls in subsequent experiments, we selected 10 mg/L 6-BA (B3) for further research. Under deep-sowing conditions, the combined treatment of exogenous SA–6-BA inhibited seedling growth, indicating that there may be an interaction between exogenous SA and 6-BA (Figure 1K–O). Compared with SA alone, the root length, seedling length, mesocotyl length and the sum of the mesocotyl and coleoptile lengths of Zi330 were significantly reduced after SA–6-BA application. However, compared with the application of 6-BA alone, the root length, seedling length and mesocotyl length of Zi330 showed an increasing trend, and the mesocotyl length increased most significantly under the C3 treatment. According to the average value of the membership function, we used C3 as the optimal concentration of the exogenous SA–6-BA combined treatment (Table S3).

### 2.2. Effects of Optimal Concentration of Exogenous SA, 6-BA and SA–6-BA on Maize Inbred Lines with Different Tolerance to Deep Sowing

#### 2.2.1. The Growth Index Changes in Maize Inbred Lines under the Optimum Concentration of Exogenous SA, 6-BA and SA–6-BA

**Figure 2:**
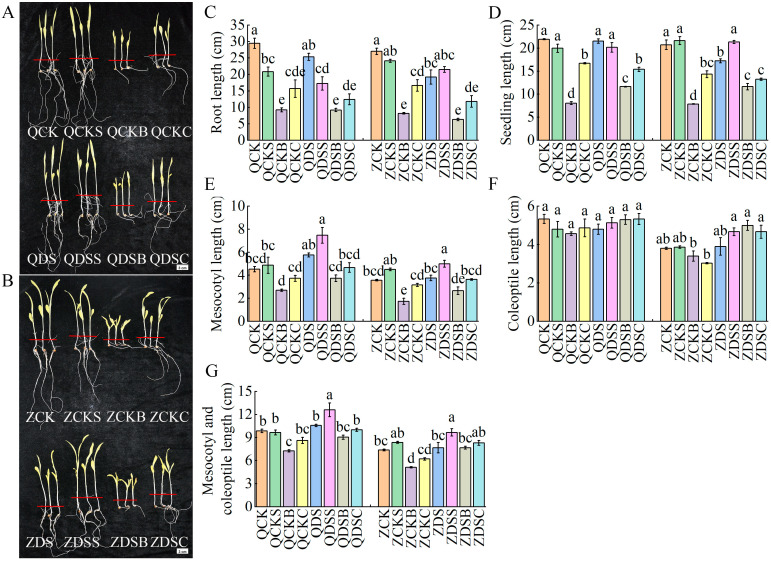
Morphological changes in maize inbred lines treated with optimal concentration of exogenous hormones. The seedling morphology of (**A**) Qi319 and (**B**) Zi330. The scale bars are 3 cm. (**C**–**G**) Phenotypic indexes of two inbred lines under different treatments. Different lowercase letters represent significant differences between treatments by Tukey’s test (*p* < 0.05).

Compared with the normal sowing depth, the mesocotyl length and the sum of the mesocotyl and coleoptile lengths of the deep-sowing tolerant inbred line Qi319 and the deep-sowing sensitive inbred line Zi330 increased significantly under deep-sowing conditions, and the increase in Qi319 was greater than that of Zi330 (Figure 2A,B). The application of exogenous SA significantly increased the mesocotyl length and the sum of the mesocotyl and coleoptile lengths under deep-sowing conditions. The application of exogenous 6-BA and SA–6-BA significantly reduced the root length, seedling length, mesocotyl length and the sum of the mesocotyl and coleoptile lengths of the two inbred lines, but the inhibitory effect of 6-BA alone was more significant (Figure 2C–G).

#### 2.2.2. Cytological Observation of Maize Mesocotyl under the Optimum Concentration of Exogenous SA, 6-BA and SA–6-BA Treatment

**Figure 3:**
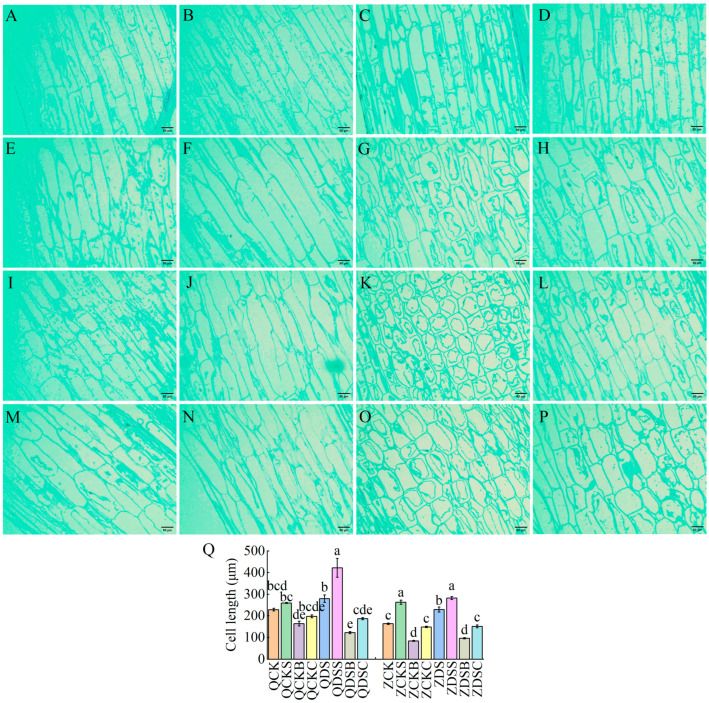
Cytological observation of maize mesocotyl under different treatments. (**A**–**H**) is the longitudinal structure of mesocotyl cells of QCK, QCKS, QCKB, QCKC, QDS, QDSS, QDSB and QDSC. The longitudinal structure of mesocotyl cells in ZCK, ZCKS, ZCKB, ZCKC, ZDS, ZDSS, ZDSB and ZDSC was in turn (**I**–**P**). The scale bars of (**A**–**P**) are all 50 μm. (**Q**) Length of cells in the middle of the mesocotyl. Different lowercase letters represent significant differences between treatments by Tukey’s test (*p* < 0.05).

In order to understand the effect of mesocotyl cell shape on the length of the mesocotyl, the mesocotyl was divided into upper, middle and lower parts, and the longitudinal growth of cells in the middle of the mesocotyl was observed by paraffin section (Figure 3A–P). Compared with the normal sowing depth, the mesocotyl cells of Qi319 and Zi330 were significantly elongated under deep-sowing conditions (Figure 3Q). After applying exogenous SA, the mesocotyl cell length of the two inbred lines increased significantly under normal-sowing and deep-sowing conditions, and the increase was Qi319 > Zi330, indicating that exogenous SA promoted mesocotyl elongation by increasing mesocotyl cell length (Figure 3Q). After applying exogenous 6-BA, the mesocotyl cell length of the two inbred lines under different sowing depths was significantly reduced, and the number of cells per unit area increased, indicating that exogenous 6-BA inhibited mesocotyl elongation by reducing mesocotyl cell length (Figure 3Q). After the application of exogenous SA–6-BA, the length of the mesocotyl cells of the two inbred lines under different sowing depths was significantly lower than that of exogenous SA and significantly higher than that of exogenous 6-BA, indicating that exogenous SA could alleviate the inhibition of exogenous 6-BA on the length of mesocotyl cells to a certain extent (Figure 3Q). Therefore, we posit that the change in mesocotyl length in maize seedlings after deep sowing is mainly caused by the longitudinal extension of mesocotyl cells.

### 2.3. Transcriptome Analysis of Maize Mesocotyls Treated with Different Exogenous Hormones under Two Sowing Depths

#### 2.3.1. RNA-seq Data Statistics and Comparison

In this study, RNA-seq was performed on mesocotyl samples collected from the maize inbred lines Qi319 and Zi330 after applying exogenous SA, 6-BA, and SA–6-BA at two sowing depths. The total RNA of 48 mesocotyl samples had high purity and good integrity, which met the requirements of database construction, and could be sequenced (Table S4). The original data were quality controlled and produced 322.12Gb of clean data. Each sample yielded 6.02Gb of clean data, with Q30 base percentages ranging from 92.3% to 93.9% and GC content ranging from 50.5% to 54.3% (Table S5). The obtained clean data were compared with the reference genome (Zm_B73_REFERENCE_NAM_5.0.new), respectively, for a fast and accurate comparison, and the efficiency of the clean reads of the 48 samples compared with the reference genome ranged from 77.8% to 89.1% (Table S6).

#### 2.3.2. Statistical Screening of DEGs

**Figure 4:**
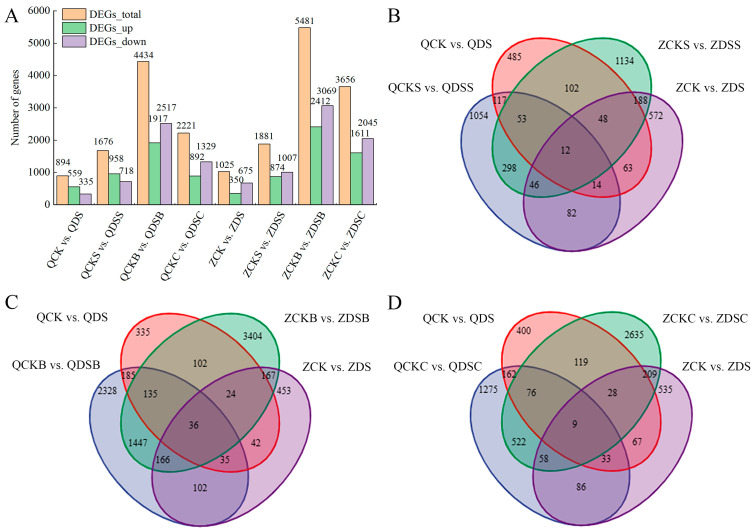
DEGs of two inbred lines under different treatments. (**A**) The number of DEGs between different groups. (**B**–**D**) Wayne diagram of DEGs between SA, 6-BA and SA–6-BA treatments and their respective control groups.

In order to further study the deep-sowing tolerance of maize and analyze the response mechanism of different maize inbred lines to exogenous hormones under different sowing-depth conditions, DEGs were screened with a fold change ≥ 2 and *p*-value < 0.05 as the standards in this study. Under deep-sowing conditions, 894 DEGs were identified in the deep-sowing tolerant inbred line Qi319 compared to the normal-sowing depth. Among these genes, 559 were up-regulated, and 335 were down-regulated. Similarly, in the deep-sowing sensitive inbred line Zi330, a total of 1025 DEGs were identified of which 350 were up-regulated and 675 were down-regulated (Figure 4A). After treatment with exogenous SA, Qi319 found 1676 DEGs under deep-sowing conditions when compared to the normal-sowing depth; a total of 958 of these genes were up-regulated, and 718 were down-regulated. Additionally, 1881 DEGs were identified from Zi330, with 874 being up-regulated and 1007 down-regulated (Figure 4A). After applying exogenous 6-BA, 4434 DEGs were identified in Qi319 under deep-sowing conditions compared to the normal-sowing depth. Among those genes, 1917 were up-regulated, and 2517 were down-regulated. Zi330 exhibited 5481 DEGs, among which 2412 were up-regulated and 3069 were down-regulated (Figure 4A). After applying SA–6-BA, a total of 2221 DEGs were identified in Qi319 at two different sowing depths. Out of these, 892 genes were up-regulated, and 1329 genes were down-regulated. A total of 3656 genes displaying differential expression were identified from Zi330, with 1611 genes up-regulated and 2045 genes down-regulated (Figure 4A). The Venn diagram showed the number of common or unique DEGs in different comparison groups. After applying exogenous SA, 6-BA and SA–6-BA, the number of DEGs shared by the two inbred lines and their respective controls was 12, 36 and 9, respectively (Figure 4B–D). In conclusion, there are some differences in the response of the two inbred lines to different treatments. Exogenous SA, 6-BA and SA–6-BA affect mesocotyl elongation by up-regulating or down-regulating more DEGs.

#### 2.3.3. Gene Ontology (GO) Enrichment Analysis of DEGs

**Figure 5:**
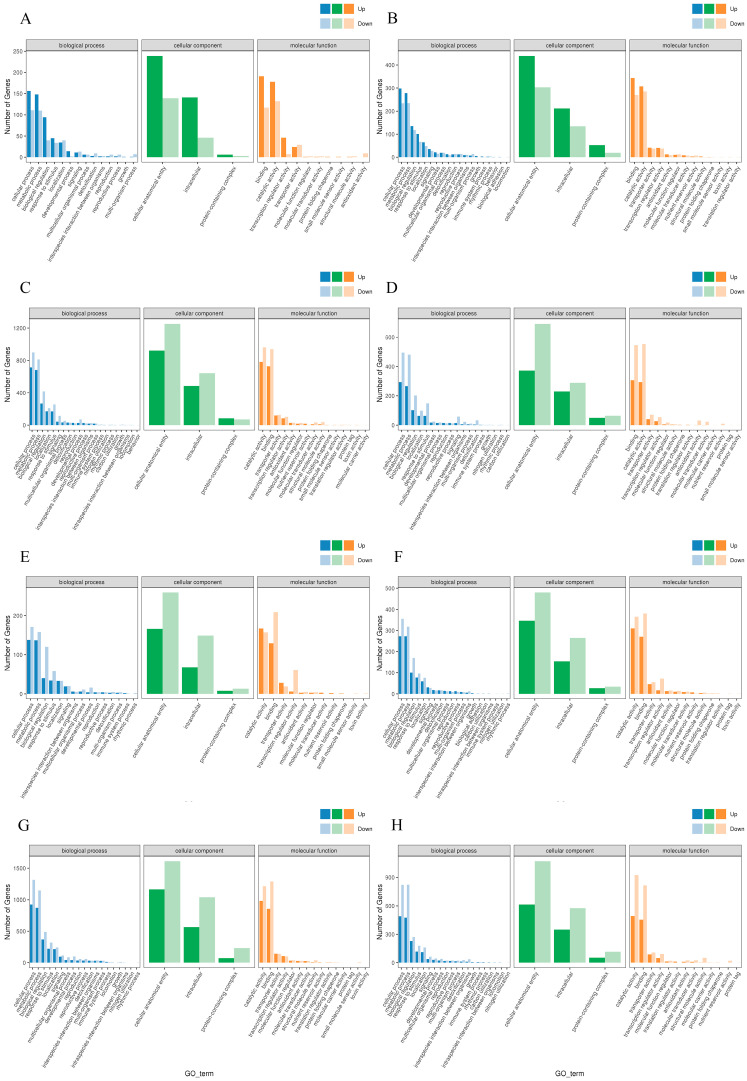
GO annotation of DEGs under different treatments of two inbred lines. (**A**) QCK vs. QDS; (**B**) QCKS vs. QDSS; (**C**) QCKB vs. QDSB; (**D**) QCKC vs. QDSC; (**E**) ZCK vs. ZDS; (**F**) ZCKS vs. ZDSS; (**G**) ZCKB vs. ZDSB; (**H**) ZCKC vs. ZDSC.

In order to explore the distribution characteristics of DEGs in response to different exogenous hormones in different maize varieties with deep-sowing tolerance, GO functional enrichment analysis was performed on the identified DEGs to determine their important biological functions. Compared with the normal-sowing depth, the main enriched biological processes (BPs) of the annotated genes in Qi319 under deep-sowing conditions were cellular process, metabolic process, biological regulation, response to stimulation, localization, signal and development process. The main enriched molecular functions (MFs) were binding, catalytic activity, transcriptional regulatory factor activity and transport activity. The main enriched cellular components (CCs) were cell anatomical entities, intracellular and protein-containing complexes (Figure 5A). The main enriched GO terms of the annotated genes obtained by Zi330 were basically the same as those of Qi319, but Zi330 had more DEGs in the same enriched term, and the number of down-regulated genes in biological regulation, cell anatomical entity, intracellular, binding and transcriptional regulatory factor activity was significantly more than the number of up-regulated genes (Figure 5E). Therefore, it is hypothesized that the variance in gene expression in these procedures could be a potential cause for the contrasting levels of deep-planting tolerance observed in the two inbred lines. After applying exogenous SA, 6-BA and SA–6-BA, we observed that the enriched gene annotations obtained from Qi319 and Zi330 were consistent with the above enrichment process. However, we found that more differential genes were expressed in the same enrichment term (Figure 5B–D,F–H), indicating that exogenous hormones may regulate mesocotyl length by regulating more differential genes. In addition, under SA treatment, Zi330 had fewer up-regulated genes and more down-regulated genes than Qi319 in the same enrichment term (Figure 5B,F); under the treatment of 6-BA and SA–6-BA, the number of up- and down-regulated genes in each enrichment item of Zi330 was greater than that of Qi319, indicating that Zi330 was more sensitive to exogenous hormones (Figure 5C,D,G,H).

#### 2.3.4. Kyoto Encyclopedia of Genes and Genomes (KEGG) Enrichment Analysis of DEGs

**Figure 6:**
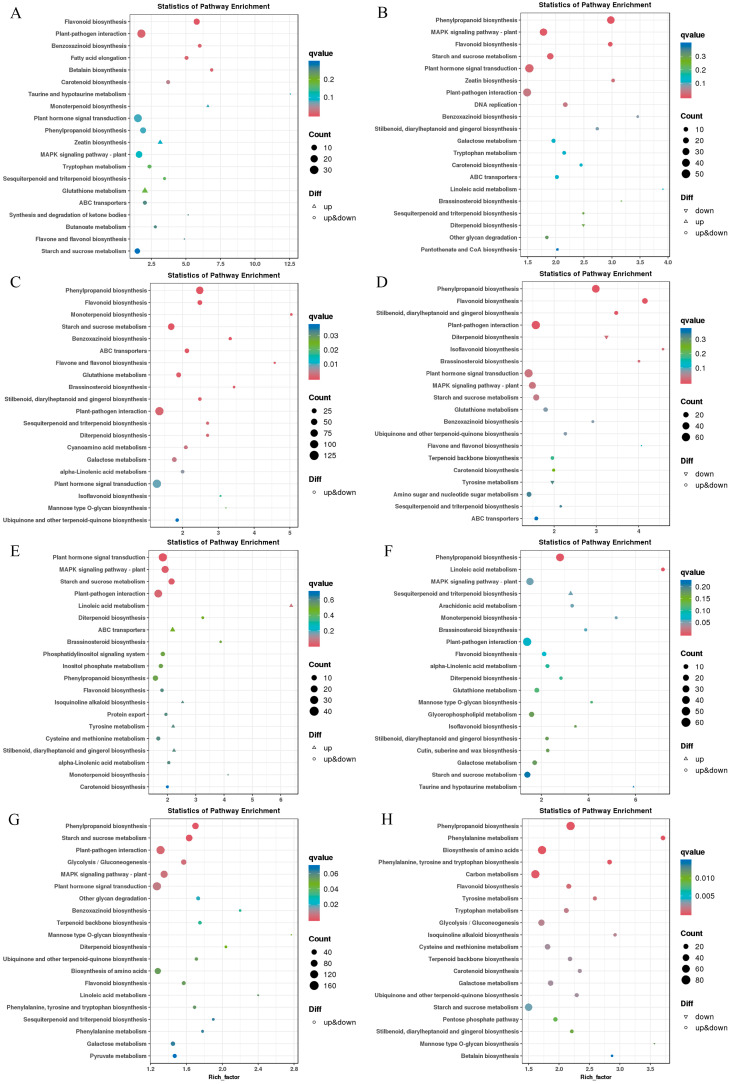
KEGG annotation of DEGs under different treatments of two inbred lines. (**A**) QCK vs. QDS; (**B**) QCKS vs. QDSS; (**C**) QCKB vs. QDSB; (**D**) QCKC vs. QDSC; (**E**) ZCK vs. ZDS; (**F**) ZCKS vs. ZDSS; (**G**) ZCKB vs. ZDSB; (**H**) ZCKC vs. ZDSC.

In order to further understand the function of DEGs, KEGG functional annotation was performed on the identified DEGs, and the first 20 pathways with the smallest significant q-value were selected for KEGG enrichment analysis. In the QCK vs. QDS comparison group, the main metabolic pathways enriched by DEGs were plant–pathogen interaction, flavonoid biosynthesis, fatty acid elongation, carotenoid biosynthesis, benzoxazine biosynthesis and betaine biosynthesis. In the ZCK vs. ZDS comparison group, the main metabolic pathways enriched by DEGs included plant hormone signal transduction, plant–pathogen interaction, MAPK signaling pathway-plant, starch and sucrose metabolism and linoleic acid metabolism. These different enrichment pathways may be related to the different responses of the two inbred lines to deep sowing (Figure 6E).

Under exogenous SA treatment, the main DEGs-enriched pathways in the QCKS vs. QDSS comparison group were plant hormone signal transduction, plant–pathogen interaction, phenylpropanoid biosynthesis, MAPK signaling pathway-plant, starch and sucrose metabolism and flavonoid biosynthesis (Figure 6B). In the ZCKS vs. ZDSS comparison group, the DEGs were mainly enriched in the pathways of phenylpropanoid biosynthesis and linoleic acid metabolism (Figure 6F). In the phenylpropanoid biosynthesis pathway, the two inbred lines mainly responded to the regulation of mesocotyl length by exogenous SA through the differential expression of cinnamyl alcohol dehydrogenase, peroxidase, cinnamoyl-CoA reductase, coniferyl aldehyde dehydrogenase and shikimate o-hydroxycinnamoyl transferase genes (Table S7).

Under exogenous 6-BA treatment, both inbred lines were enriched in plant hormone signal transduction, plant–pathogen interaction, phenylpropanoid biosynthesis and starch and sucrose metabolism pathways, indicating that exogenous 6-BA may affect the growth of maize mesocotyls by regulating the expression of DEGs in these pathways (Figure 6C,G). In addition, Qi319 was also enriched in glutathione metabolism, flavonoid biosynthesis, ABC transporters, galactose metabolism and cyanoamino acid metabolism pathways. Zi330 was also enriched in MAPK signaling pathway-plant and the glycolysis/gluconeogenesis process (Figure 6C,G). The enrichment of the plant hormone signal transduction pathway showed that the genes responding to exogenous 6-BA were involved in the synthesis and metabolism of IAA, BR, JA and ETH. In addition, MYC2 (myelocytomatosis 2) transcription factor, DELLA protein, phytochrome interacting factor 4 (PIF4), two-component response regulator ARR-B family and some protein kinases were also involved in the negative regulation of exogenous 6-BA on maize mesocotyl length (Table S8).

After the application of exogenous SA–6-BA, in the QCKC vs. QDSC comparison group, DEGs were enriched in plant–pathogen interaction, plant hormone signal transduction, phenylpropanoid biosynthesis, MAPK signaling pathway-plant, starch and sucrose metabolism and the flavonoid biosynthesis pathway (Figure 6D). In the ZCKC vs. ZDSC comparison group, DEGs were enriched in phenylpropanoid biosynthesis, amino acid biosynthesis, carbon metabolism, phenylalanine metabolism, phenylalanine, tyrosine and tryptophan biosynthesis and flavonoid biosynthesis pathways (Figure 6H). It indicated that phenylpropanoid biosynthesis and flavonoid biosynthesis were the main pathways for the interaction of exogenous SA and 6-BA to regulate the length of maize mesocotyl.

### 2.4. RT-qPCR Validation

**Figure 7:**
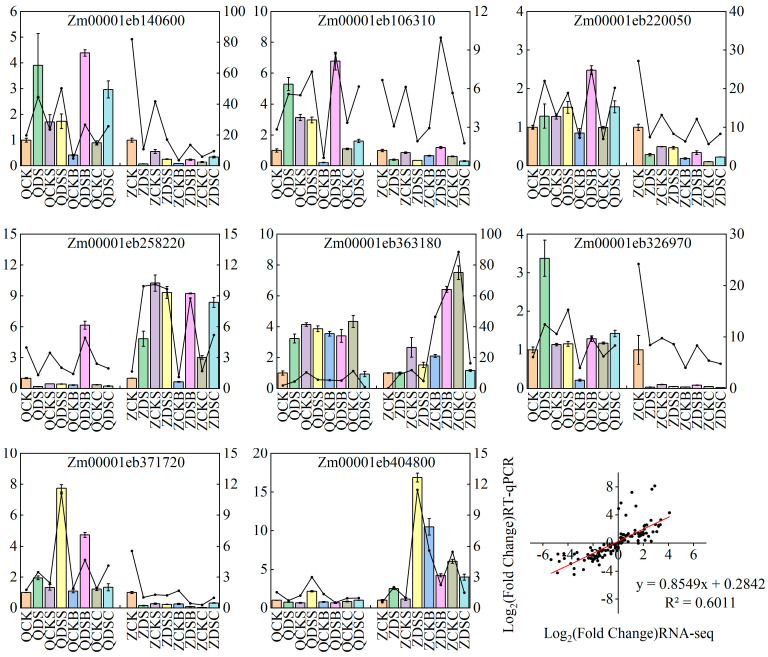
RT-qPCR of DEGs. Bar charts and line charts represent RT-qPCR and RNA-seq data, respectively.

In order to further verify the reliability of the transcriptome sequencing results, we randomly selected eight DEGs for RT-qPCR analysis. The relative expression trend of these genes is consistent with the RNA-seq results (Figure 7), indicating that our sequencing results are reliable.

### 2.5. Weighted Gene Co-Expression Network Analysis (WGCNA) Analysis

#### 2.5.1. Construction of Gene Co-Expression Module Based on WGCNA

**Figure 8:**
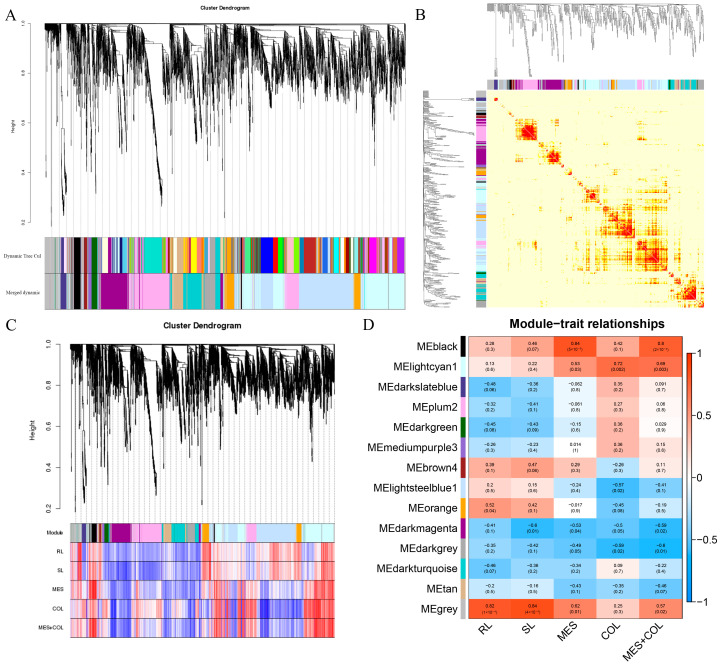
Module construction based on WGCNA. (**A**) Gene network module. (**B**) Gene co-expression network heat map. (**C**) Gene phylogenetic tree and trait correlation heat map. (**D**) Heatmap of correlation between modules and traits. The closer the correlation is to the absolute value of 1, the more relevant the trait is to the gene of the module.

WGCNA can find co-expressed gene modules by constructing a co-expression network, explore the correlation between co-expressed gene modules and sample traits and mine core genes highly associated with traits. In order to clarify the correlation between the genes obtained by RNA-seq and the phenotypic data of maize deep sowing, we constructed a gene co-expression network through WGCNA and identified 14 different co-expression modules (Figure 8A,B). Among them, root length was significantly correlated with the MEgrey and MEorange modules; seedling length was significantly correlated with the MEgrey module; mesocotyl length was significantly correlated with the MEblack, MEgrey and MElightcyan1 modules; and coleoptile length was significantly correlated with the MElightcyan1 module. The sum of the mesocotyl and coleoptile lengths was significantly correlated with the MEblack, MElightcyan1 and MEgrey modules (Figure 8C,D).

#### 2.5.2. Gene Function Analysis of Related Modules

**Figure 9:**
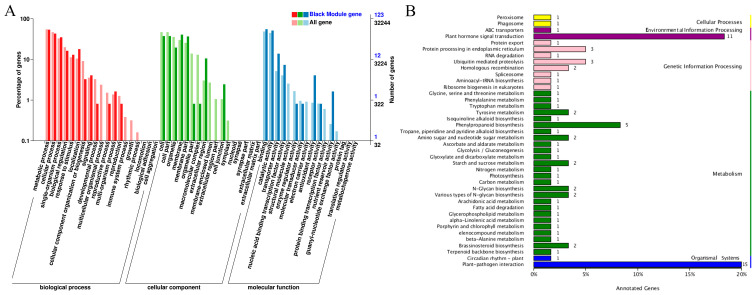
Functional analysis of genes in the black module. (**A**) GO enrichment analysis. (**B**) KEGG enrichment analysis.

In order to further study the function of mesocotyl-related genes, GO and KEGG were used to analyze the genes in the black module with the highest correlation with mesocotyl length. The module gene GO enriched BPs include metabolic processes, cellular processes, single organism processes, localization, biological regulation and response to stimuli. The enriched CCs include membrane, cell, cell part, membrane part and organelle. The enriched MFs include binding, catalytic activity and transport activity (Figure 9A). The KEGG metabolic pathways are mainly plant hormone signal transduction, plant–pathogen interaction, phenylpropanoid biosynthesis, protein processing in endoplasmic reticulum and ubiquitin-mediated proteolysis (Figure 9B).

#### 2.5.3. Co-Expression Network Visualization

**Figure 10:**
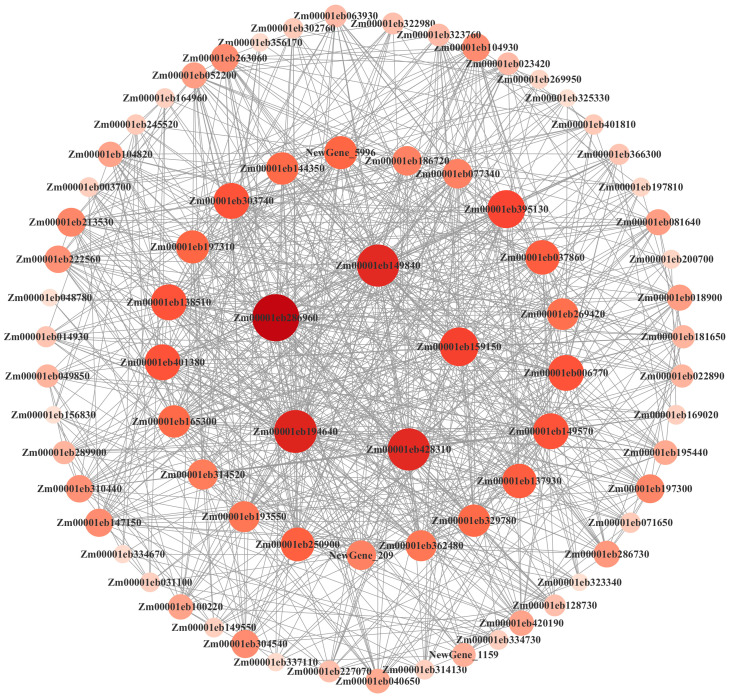
Interaction network analysis of hub genes in the black module. The size and color of points represent the level of gene connectivity.

Hub genes are usually key regulatory genes, which are preferentially used for in-depth analysis and mining. We used the MEblack module to show the network of the top 77 genes with a connectivity greater than 10 (Figure 10). The five genes with the highest connectivity were Zm00001eb286960, Zm00001eb149840, Zm00001eb194640, Zm00001eb159150 and Zm00001eb428310. Gene annotations were BRCA1-A complex subunit BRE, NA (no annotation), auxin efflux carrier family protein, uroporphyrin-III C-methyltransferase and ATP-binding cassette and subfamily C, respectively. Therefore, we predicted that auxin-activated signaling pathways, transmembrane transport, methylation and redox processes may be involved in the regulation of maize mesocotyl growth (Table S9).

## 3. Discussion

### 3.1. Effects of Deep Sowing and Exogenous Hormones on the Growth of Maize Seedlings

Deep sowing is an important measure to alleviate drought. After deep sowing, crops can absorb water from deep soil to ensure sufficient water near the seeds to promote germination and emergence. At the same time, the root system of maize is deeply distributed after deep sowing, which can enhance the lodging resistance of the plants [21]. Studies have reported that Indian Blue Kernel Corn has the characteristics of extending rhizomes and pushing germs to suitable soil layers to achieve seedling emergence under the condition of a sowing depth of 20~25 cm [22]. The deep-sowing tolerance of maize is related to the emergence rate, seedling length, root length, mesocotyl length, coleoptile length and the sum of the mesocotyl and coleoptile lengths [23], and the mesocotyl is the main organ for maize seedlings to adapt to the deep-sowing environment [4]. In this study, the root length, seedling length, mesocotyl length, coleoptile length and the sum of the mesocotyl and coleoptile lengths of the maize inbred lines Qi319 and Zi330 under deep-sowing conditions all changed to varying degrees, among which mesocotyl elongation was the most significant. Studies have shown that exogenous IAA [24], GA_3_ [25], BR, SL (strigolactone) [26], EBR (2,4-Epibrassinolide Solution) [27], etc., promote the elongation of mesocotyls under deep-sowing conditions. In this study, we found that exogenous SA significantly promoted the elongation of the mesocotyl, exogenous 6-BA significantly inhibited the elongation of the mesocotyl, and the SA–6-BA combined treatment also showed an inhibitory effect, but the inhibitory effect was weaker, indicating that there may be an interaction between these two hormones; exogenous SA partially alleviated the inhibitory effect of exogenous 6-BA on mesocotyl elongation. This study visually observed the changes in mesocotyl cell length through paraffin sections. The results showed a significant increase in cell length in the middle of the mesocotyl for both inbred lines under deep-sowing conditions. The impact of exogenous hormones on the cells in the middle of the mesocotyl was consistent with the phenotypic changes observed, which aligns with the findings of previous studies [26]. This suggests that deep sowing or exogenous hormones can influence mesocotyl growth by regulating the longitudinal elongation of mesocotyl cells, ultimately affecting seedling emergence.

### 3.2. Mechanism of Exogenous SA Regulating the Length of Maize Mesocotyl

**Figure 11:**
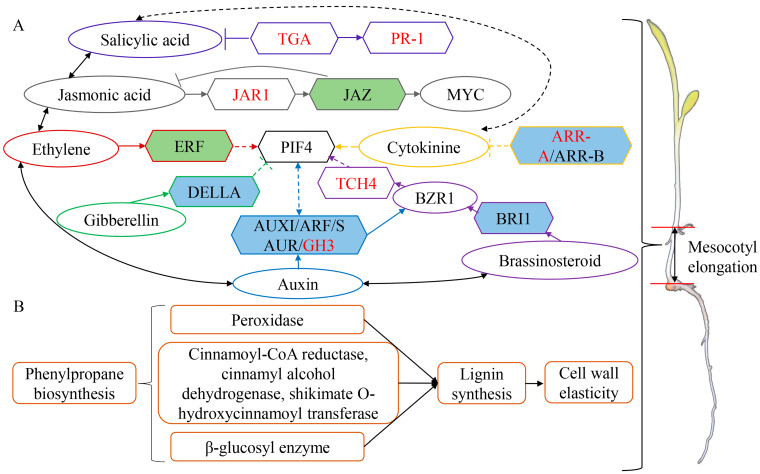
Plant hormone signal transduction pathway and phenylpropanoid biosynthesis pathway related to mesocotyl elongation. (**A**) Key nodes of plant hormone signaling pathway. The green box represents the node that responds to exogenous SA, the blue box represents the node that responds to exogenous 6-BA, and the red font represents the node that responds to exogenous SA–6-BA. TGA: TGACG motif binding protein; PR-1: pathogenesis-related protein-1; JAR-1: jasmonic acid resistant 1; JAZ: Jasmonate ZIM-domain; MYC: myelocytomatosis; ERF: ethylene responsive factor; PIF4: phytochrome interacting factor 4; ARR-A: type-A response regulators; ARR-B: type-B response regulators; DELLA: aspartic acid–glutamic acid–leucine–leucine–alanine; AUXI: Auxin/indole-3-acetic acid; ARF: auxin response factor; SAUR: small auxin-up RNA; GH3: Gretchen Hagen 3; BZR1: brassinazole resistant 1; BRI1: brassinosteroid insensitive 1; TCH4: xyloglucan endotransglucosylase/hydrolase (XTH) family member. (**B**) Phenylpropanoid biosynthesis pathway.

The effect of exogenous SA on plants is related to the application method, action time and plant development stage, and the effective concentration varies from species to species [28,29]. In this study, different concentrations of exogenous SA were applied to different maize inbred lines with two sowing depths. It was found that a high concentration of SA inhibited the growth of maize seedlings, which may be due to the oxidative damage of plants caused by a high concentration of SA and the accumulation of H_2_O_2_ leading to cell death [30]. However, low concentrations of exogenous SA promoted the growth of maize seedlings, and 0.25 mmol/L SA had the best effect on the promotion of maize mesocotyls. Transcriptome analysis showed that, compared with the control, the two inbred lines had more DEGs after the application of exogenous SA. The JAZ (jasmonic acid ZIM-domain) protein and ERF (ethylene response transcription factor) gene in the DEGs shared by the two inbred lines were up-regulated in Qi319 and down-regulated in Zi330 (Table S10). This may be due to the different response mechanisms of maize inbred lines with different deep-sowing tolerance to exogenous SA. It has been reported that JAZ protein is a negative regulator of the JA signaling pathway [31]. JA and ETH have antagonistic effects. ETH reduces JA levels by inhibiting the expression of genes such as GY1 in the JA biosynthesis pathway to promote the growth of mesocotyls and coleoptiles in rice [16]. In Qi319, the up-regulated expression of JAZ protein and ETH responsive transcription factor ERF gene may promote the growth of the mesocotyl by inhibiting the JA signaling pathway. However, studies have also found that JA and ethylene have a synergistic effect [32]. We predicted that the down-regulated expression of the ERF gene in Zi330 may also promote the growth of mesocotyls by inhibiting the JA signaling pathway. At the same time, due to the sensitivity of Zi330, the hormone signals induced by exogenous SA may reach saturation. In order to maintain the balance of plant growth and development, the signals are coordinated and down-regulated to promote the growth of mesocotyls. In addition, shikimate o-hydroxycinnamoyl transferase can affect cell wall growth elasticity by regulating lignin content [33]. The up-regulated expression of the shikimate o-hydroxycinnamoyl transferase gene in the two inbred lines (Table S10) may promote the elongation of mesocotyl cells by increasing cell wall elasticity. The up-regulated expression of cytochrome P450 family genes (Table S10) may be involved in plant growth regulation through various synthetic pathways, such as phenylpropanoids, alkaloids, terpenoids and phytohormones [34]. Therefore, the JA signaling pathway, ETH signaling pathway, cytochrome P450 family genes and lignin synthesis related genes are involved in the growth of the mesocotyl regulated by SA signaling (Figure 11A).

### 3.3. Mechanism of Exogenous 6-BA Regulating the Length of Maize Mesocotyl

This study found that exogenous 6-BA inhibited the growth of maize seedlings, and the inhibitory effect increased with the increase in concentration. Transcriptomic analysis revealed the identification of 4434 DEGs in Qi319 with exogenous 6-BA treatment, 56.77% of which were down-regulated. Similarly, 5481 DEGs were identified in Zi330, with 55.99% showing down-regulation. These findings suggest that 6-BA may inhibit mesocotyl elongation by repressing a considerable number of cell growth-related genes. It has been discovered that the polar transportation of IAA is crucial for mesocotyl growth [35]. The down-regulated expression of a large number of auxin-related genes (Zm00001eb135570, Zm00001eb147480, Zm00001eb292830, Zm00001eb433460, Zm00001eb133000, Zm00001eb232120, Zm00001eb135550, Zm00001eb191370, Zm00001eb284310, Zm00001eb408800, Zm00001eb066640, Zm00001eb350370) in the two inbred lines may inhibit the growth of the mesocotyl (Table S8). It was reported that spraying exogenous 6-BA in the dark inhibited the hypocotyl elongation of Picea crassifolia Kom [36], and 6-BA inhibited the hypocotyl elongation of Arabidopsis thaliana in the dark by interacting with IAA and ETH [37]. In addition, under light conditions, ETH promotes hypocotyl elongation by activating PIF3 (phytochrome interacting factor 3) through EIN3 (ethylene insensitive 3). Under dark conditions, ETH can enhance the stability of ERF1 to inhibit hypocotyl elongation [38]. Therefore, the inhibition of maize mesocotyl elongation by 6-BA in this study may be related to dark conditions. PIF4 is a positive regulator of cell elongation. PIF4 inhibits photomorphogenesis in the dark together with other PIF members [39]. Under 6-BA treatment, the down-regulated expression of phytochrome-related genes (Zm00001eb059460, Zm00001eb332400) may inhibit cell elongation (Table S8). GA-induced degradation of DELLA protein can promote the accumulation of PIFs protein [40]. PIFs activate the expression of auxin synthesis gene YUCs and auxin signaling genes AUX/IAA and ARFs (auxin response factors) by transcription, promote the accumulation of auxin transport protein PIN (pin-formed), increase the concentration of auxin in cells and promote plant cell elongation [41]. The up-regulated expression of DELLA protein genes (Zm00001eb187310, Zm00001eb082700, Zm00001eb164480, Zm00001eb164530, Zm00001eb320950) may affect the expression of auxin-related genes by inhibiting the accumulation of PIFs protein, thereby inhibiting mesocotyl elongation (Table S8). BR is involved in the induction of plant hypocotyl elongation by regulating the transcriptional activity of BZR1 (brassinazole resistant 1) and BES1 (bri1-ems suppressor 1) [42]. BZR1 can directly interact with PIF4 protein, enhance PIF4 activity and promote plant hypocotyl elongation [43]. Auxin response factor ARF6 can interact with PIF4 and BZR1 to synergistically regulate downstream target genes, thereby promoting plant hypocotyl elongation [44]. Therefore, we predicted that the hormone–protein interaction regulatory network mediated by PIF4 is involved in the negative regulation of mesocotyl growth under dark conditions. In addition, the up-regulated expression of JAZ protein genes (Zm00001eb084980, Zm00001eb223590) may affect mesocotyl growth by inhibiting the JA signaling pathway (Table S8). Type-B ARRs (type-B response regulators) have transcriptional activation activity and play a positive role in regulating CTK signal transduction by promoting the transcription of cytokinin-responsive genes [45]. The down-regulated expression of two-component response regulator ARR-B family genes (Zm00001eb030400, Zm00001eb233310, Zm00001eb144290, Zm00001eb227200, Zm00001eb365420, Zm00001eb389770) may inhibit this signal transduction process (Table S8). In conclusion, IAA, PIF, DELLA protein, BR, JAZ protein and two-component response regulator ARR-B family related genes may play an important role in the growth of the mesocotyl (Figure 11A).

### 3.4. Regulation of Exogenous SA and 6-BA Interaction on the Length of Maize Mesocotyl

The crosstalk between hormones has an important influence on plant growth and development. This study found that SA–6-BA treatment significantly reduced the root length, seedling length, mesocotyl length and the sum of the mesocotyl and coleoptile lengths of Qi319 and Zi330, but the inhibitory effect on mesocotyl length was weaker than that of 6-BA alone. We predicted that this inhibition is due to the interaction between exogenous SA and 6-BA. The analysis of the transcriptome revealed that the interaction between SA and 6-BA inhibited the expression of genes belonging to the GH3 family in the IAA signaling pathway, the JAR1 (jasmonic acid resistant 1) gene in the JA signaling pathway, the ARR-A (type-A response regulators) family genes in the CTK signaling pathway and the PR-1 (pathogenesis-related protein 1) gene in the SA signaling pathway (Figures S1–S4). The GH3 (gretchen hagen 3) protein is capable of catalyzing amino acids to form conjugates with free IAA, JA and SA [46]. JAR, a class I GH3 protein member, catalyzes the combination of JA and isoleucine (Ile) to generate jasmonic acid isoleucine (JA-Ile). JA-Ile promotes the interaction between COI1 (coronatine insensitive 1) and JAZ, leading to JAZ degradation by the 26S proteasome, thereby activating transcription factors such as MYC and MYB, directly regulating JA response genes and, subsequently, impacting cell growth [47]. Type-A ARR is the primary response gene of CTK, and its response is regulated by type-B ARR [48]. Takatoshi et al. [49] found that the expression of ARR4~ARR9, ARR15 and ARR16 in roots and leaves of wild-type Arabidopsis thaliana was significantly enhanced after treatment with CTK (20 μmol/L t-zeatin). In this study, the down-regulated expression of two-component response regulator ARR-A family genes under SA–6-BA treatment may be due to the inhibition of the role of 6-BA by SA-induced signal nodes. The expression of the defense gene PR-1 is regulated by NPR1 (nonexpressor of pathogenesis-related genes 1). As a receptor of SA, NPR1 can bind to the TGA (TGACG motif binding protein) transcription factor dimer to form a complex, thereby inducing transcription [50]. Under SA–6-BA treatment, the down-regulated expression of NPR1 and TGA in Qi319 may inhibit the expression of the PR-1 gene by inhibiting the transcriptional activation process. However, the expression of the PR-1 gene also depends on the members of the TGA transcription factor family, and TGA transcription factors negatively control the accumulation of SA [51]. Under SA–6-BA treatment, the up-regulated expression of TGA in Zi330 may inhibit the accumulation of SA, which in turn affects the binding of NPR1 to SA, resulting in the inhibition of mesocotyl elongation. In addition, in this study, the BR signaling pathway TCH4 was down-regulated in Qi319 and up-regulated in Zi330 (Figure S5). It has been reported that TCH4 can regulate cell wall homeostasis, and the expression level of TCH4 can be rapidly up-regulated by BR, IAA and various environmental stimuli (such as touch, temperature and darkness) [52]. In summary, the interaction between exogenous SA and 6-BA exists between multiple plant hormone signaling nodes, which inhibits the growth and development of mesocotyls by affecting signal transduction, gene expression and metabolite synthesis (Figure 11A).

### 3.5. The Main Metabolic Pathways of DEGs

This study found that, under different sowing depths, DEGs in mesocotyls were mainly enriched in plant hormone signal transduction, plant–pathogen interaction, MAPK signaling pathway-plant, starch and sucrose metabolism and the phenylpropanoid biosynthesis pathway (Figure 6A,E), which was basically consistent with the results of Wang et al. [53] and Leng et al. [25]. The phenylpropanoid biosynthesis pathway was significantly enriched in Qi319 and Zi330 under the interaction of exogenous SA, 6-BA and SA–6-BA (Figure 6). Therefore, the phenylpropanoid biosynthesis pathway may be the key regulatory pathway of maize mesocotyl in response to exogenous SA and 6-BA signals. There were 12, 59 and 28 DEGs shared by the two inbred lines in the phenylpropanoid biosynthesis pathway under the interaction of exogenous SA, 6-BA and SA–6-BA (Tables S7, S11 and S12), mainly including genes related to cinnamoyl-CoA reductase, cinnamyl alcohol dehydrogenase, shikimate o-hydroxycinnamoyl transferase, peroxidase and *β*-glucosidase. Cinnamoyl-CoA reductase and cinnamyl alcohol dehydrogenase are the key enzymes in the lignin biosynthesis pathway. The expression of the cinnamoyl-CoA reductase gene was down-regulated under the treatment of exogenous SA, 6-BA and SA–6-BA, which may lead to the decrease in lignin content and the accumulation of monolignol precursors [54]. However, under the treatment of exogenous SA and 6-BA, the expression of the cinnamyl alcohol dehydrogenase gene was up-regulated and down-regulated, while the expression was down-regulated under the treatment of exogenous SA–6-BA. Cinnamyl alcohol dehydrogenase and cinnamoyl-CoA reductase are closely related, and the coordination of the two plays an important role in regulating the phenylalanine metabolic pathway to lignin monomer synthesis [54]. The shikimate o-hydroxycinnamoyl transferase gene is up-regulated under SA treatment, both up-regulated and down-regulated under 6-BA treatment and down-regulated under SA–6-BA treatment. Shikimate o-hydroxycinnamoyl transferase also affects the synthesis and content of lignin in plants, thereby affecting plant growth [55]. Studies have found that the accumulation of H_2_O_2_ induces POD to oxidize single lignans in the cell wall into free radicals and polymerize to produce lignin, causing the cell wall to harden and hinder elongation growth [56]. In this study, peroxidase-related genes were up-regulated and down-regulated under exogenous SA and 6-BA treatment and down-regulated under exogenous SA–6-BA treatment. We predicted that the differential expression of peroxidase genes may also affect cell growth by regulating lignin accumulation. The differential expression of these genes is related to the synthesis and metabolism of lignin. Lignin is the main component of the secondary cell wall. The change in lignin content and composition structure is directly related to the elasticity of the cell wall [26], and the increase in lignin content will have a negative impact on cell wall relaxation and cell elongation [57]. Therefore, the biosynthesis and metabolism of lignin play an important role in regulating the extension of maize mesocotyl cells. *β*-glucosidase belongs to cellulases. Rice Os12BGlu38 is related to the formation of the inner cell wall [58]. GH1 *β*-glucosidase may contribute to the cell expansion and division of gramineous plants [59]. Under the treatment of exogenous SA and SA–6-BA, the up-regulated expression of the *β*-glucosidase gene may be helpful to promote the growth of mesocotyl cells. Under the treatment of exogenous 6-BA, the down-regulated expression of the *β*-glucosidase gene may play a role in inhibiting cell growth. In addition, it has been reported that Os1*β*Glu4 is involved in the accumulation of flavonoids by regulating the level of free SA in cells [60]. Inhibition of the flavonoid synthesis pathway contributes to lignin synthesis [61]. Therefore, the differential expression of *β*-glucosidase genes may affect cell wall expansion or lignin metabolism. In summary, the anabolic metabolism of lignin-related secondary metabolites mediated by the phenylpropanoid biosynthetic pathway is highly correlated with the elongation of maize mesocotyl cells (Figure 11B).

## 4. Materials and Methods

### 4.1. Experiment Material

The deep-sowing tolerant inbred line Qi319 (with long mesocotyl) and deep-sowing sensitive inbred line Zi330 (with short mesocotyl) screened in the laboratory’s pre-tests were used as the experimental materials. Exogenous SA (C_7_H_6_O_3_) and 6-BA (C_12_H_11_N_5_) were purchased from Solarbio (Beijing, China).

### 4.2. Experimental Method

This experiment was carried out under the conditions of 3 cm (CK) and 15 cm (DS) sowing depths. The selection of sowing depth was based on the previous experimental basis of our research group [62]. Uniformly full seeds were selected and sterilized with 0.5% NaClO solution for 10 min, rinsed with distilled water 3–5 times and then soaked in distilled water for 12 h. Firstly, the vermiculite, uniformly prepared with distilled water or hormone solutions of different concentrations (vermiculite and the corresponding treatment solution ratio of 5 g:1 mL), was spread on the bottom of the deep-sowing containers (height 25 cm, diameter 12 cm.) to a height of 10 cm. Next, the pre-soaked seeds were uniformly sown on the vermiculite with 10 seeds per pot. Then, the seeds were covered with 3 cm or 15 cm height of vermiculite mixed with distilled water or corresponding treatment solution, respectively. After sowing, they were placed in an intelligent artificial climate chamber for dark culture, and 50 mL of distilled water or corresponding treatment solution was quantitatively poured every 2 days. After 12 days of germination, the related traits were measured. Zi330 was used as the experimental material to screen the optimal concentration of different exogenous hormones under the condition of 15 cm sowing depth. The exogenous SA concentration gradients were set at 0.1 (S1), 0.25 (S2), 0.5 (S3), 1 (S4) and 2 mmol/L (S5). The exogenous 6-BA concentration gradients were set at 1 (B1), 5 (B2), 10 (B3), 15 (B4) and 20 mg/L (B5). SA–6-BA compound treatment was set up with the optimal concentration of selected single hormone, and the concentration gradient was set at C1: 0.25 mmol/L SA + 10 mg/L 6-BA, C2: 1/2 (0.25 mmol/L SA + 10 mg/L 6-BA), C3: 1/4 (0.25 mmol/L SA + 10 mg/L 6-BA), C4: 1/2× 0.25 mmol/L SA + 10 mg/L 6-BA and C5: 0.25 mmol/L SA + ½ × 10 mg/L 6-BA. Cytological observations and transcriptome sequencing were performed using Qi319 and Zi330 as the experimental materials, and the optimal concentration treatments of each exogenous hormone were expressed in Qi319 as QCK (3 cm + distilled water), QCKS (3 cm + 0.25 mmol/L SA), QCKB (3 cm + 10 mg/L 6-BA), QCKC (3 cm + 0.25 mmol/L SA + 10 mg/L 6-BA), QDS (15 cm + distilled water), QDSS (15 cm + 0.25 mmol/L SA), QDSB (15 cm + 10 mg/L 6-BA) and QDSC (15 cm + 0.25 mmol/L SA + 10 mg/L 6-BA), which is denoted as ZCK (3 cm + distilled water), ZCKS (3 cm + 0.25 mmol/L SA), ZCKB (3 cm + 10 mg/L 6-BA), ZCKC (3 cm + 0.25 mmol/L SA + 10 mg/L 6-BA), ZDS (15 cm + distilled water), ZDSS (15 cm + 0.25 mmol/L SA), ZDSB (15 cm + 10 mg/L 6-BA) and ZDSC (15 cm + 0.25 mmol/L SA + 10 mg/L 6-BA) in Zi330.

### 4.3. Determination of Deep Sowing-Related Indexes and Comprehensive Evaluation of Deep-Sowing Tolerance

The growth indices of mesocotyl length (MES), coleoptile length (COL), seedling length (SL) and root length (RL) of maize seedlings were determined using the method of Peng et al. [62]. The cytological structure of the middle part of the maize mesocotyl was observed via saffron solid green staining and paraffin-section technique.

The membership function method was used to comprehensively evaluate the deep-sowing tolerance of maize seedlings after applying different concentrations of exogenous hormones. The membership function calculation formula is as follows:

(1) $$U_{ij}=(X_{ij}-X_{jmin})/(X_{jmax}-X_{jmin})$$

(2) $$U_{ij}=1-(X_{ij}-X_{jmin})/(X_{jmax}-X_{jmin})$$

Among them, $U_{ij}$ represents the membership function value of the deep-sowing tolerance of the *j* index under *i* concentration; $X_{ij}$ represents the measured value of the *j* index under *i* concentration; and $X_{jmin}$ and $X_{jmax}$ are the minimum and maximum values of the measured values of the *j* index at different concentrations, respectively. If the measured index is positively correlated with the deep-sowing tolerance of the material, the membership function value is calculated using Formula (1); otherwise, Formula (2) is used. The membership function values of each index are accumulated and compared after averaging. The larger the average value, the stronger the deep-sowing tolerance.

### 4.4. RNA Extraction and Transcriptomics Analysis

#### 4.4.1. Total RNA Extraction, Library Construction and Illumina Sequencing

The mesocotyls of the two inbred lines under different treatments were quickly loaded into a freezer tube and placed in liquid nitrogen for quick freezing and then stored in a refrigerator at −80 °C for subsequent experiments. Three biological replicates were set up for the experiment. RNA prep Pure Plant Kit (Tiangen, Beijing, China) was used to extract total RNA from the mesocotyl samples, and RNA concentration and purity were detected by a NanoDrop 2000 (Thermo Fisher Scientific, Wilmington, DE, USA) spectrophotometer. RNA integrity was assessed using the RNA Nano 6000 detection kit of the Agilent Bioanalyzer 2100 system (Agilent Technologies, Santa Clara, CA, USA). The qualified total RNA of each sample was used for RNA-seq library construction. After the library quality inspection was qualified, PE150 sequencing was performed using the Illumina NovaSeq6000 sequencing platform. The sequencing was completed by Beijing BioMarker Technologies Co., Ltd. (Beijing, China).

#### 4.4.2. Quality Assessment of Sequencing Results

Unqualified sequences were removed from the raw data to obtain clean data, and Hisat2 was used to compare the clean data to the reference genome (Zm_B73_REFERENCE_NAM_5.0.new) to obtain the localization information of reads on the reference genome. Then, String Tie was used to assemble and reconstruct the transcriptome from the reads on the comparison, and FPKM (fragments per kilobase per million) was used to calculate the expression levels of the sample genes.

#### 4.4.3. Identification and Functional Annotation of Differentially Expressed Genes (DEGs)

DEGs from different comparison groups were analyzed by DESeq2; DEGs were screened with FC (fold change) ≥ 2 and *p*-value < 0.05 as the standards, and the screened DEGs were subjected to GO and KEGG enrichment analysis using the R/clusterProfiler.

#### 4.4.4. Real-Time Fluorescence Quantitative PCR (RT-qPCR) Verification of DEGs

The cDNA was synthesized using the TOYOBO Reverse Transcription Kit (TOYOBO Life Science, Shanghai, China). The reverse transcription procedure included incubation at 37 °C for 15 min, 50 °C for 5 min and 98 °C for 15 min, followed by preservation at 4 °C. The cDNA obtained from reverse transcription was stored at −20 °C for subsequent experiments. Eight DEGs in Qi319 and Zi330 were randomly selected, and their specific primers were designed by NCBI. The RT-qPCR primer sequences are presented in Table S13. The RT-qPCR employed the TB Green^®^ Premix Ex TaqTM II (Tli RNaseH Plus) kit (TaKaRa Biomedical Technology Co., Ltd. Beijing, China), with Actin serving as the internal reference gene. A 20 μL reaction system, comprising 10 μL TB Green Premix Ex Taq II, 0.4 μL ROX Reference Dye II (50×), 2 μL cDNA, 0.8 μL forward and reverse primers and 8 μL sterile water, was used. The RT-qPCR was performed on a QuantStudio5 real-time PCR system, and the relative expression of the gene was calculated using 2^−ΔΔCt^ [63].

#### 4.4.5. Weighted Gene Co-Expression Network Analysis (WGCNA)

Using the gene expression data obtained by RNA-seq, the FPKM threshold is set to 1, the module similarity threshold is 0.25, the minimum number of genes in the module is 30, and the co-expressed gene module is constructed using the WGCNA R package. The co-expression modules were associated with phenotypic traits, and genes with kME > 0.7 were selected as module members (kME is the characteristic gene connectivity for screening hub genes). Cytoscape_v3.10.1 software was used to visualize the gene interaction network of the core module. Finally, the hub genes in the core modules were determined according to the kME value and gene connectivity [64].

### 4.5. Data Statistical Analysis

Microsoft Excel 2016 was used for data statistics, IBM SPSS Statistics 21.0 software was used for one-way ANOVA (*p* < 0.05), Origin 2022 was used for statistical graphing, and all measurements were expressed as mean ± standard error.

## 5. Conclusions

This study found that 0.25 mmol/L exogenous SA promoted the growth of maize mesocotyls. Exogenous 6-BA inhibited seedling growth, mainly by inhibiting seedling length and mesocotyl length, and the inhibitory effect increased with the increase in concentration. Exogenous SA–6-BA compound treatment also inhibited the growth of mesocotyls. Cytological observation showed that exogenous SA significantly promoted the length of middle mesocotyl cells. Exogenous 6-BA significantly inhibited the length of middle mesocotyl cells. Exogenous SA–6-BA compound treatment also significantly inhibited the length of middle mesocotyl cells, but exogenous SA could alleviate the inhibitory effect of exogenous 6-BA on the length of mesocotyl cells to a certain extent. Transcriptome results showed that genes related to the JA signaling pathway, the ETH signaling pathway, the cytochrome P450 family and lignin biosynthesis were involved in the positive regulation of exogenous SA on mesocotyl growth. IAA, PIF, DELLA protein, BR, JAZ protein and two-component response regulator ARR-B family were involved in the negative regulation of exogenous 6-BA on mesocotyl growth. Exogenous SA–6-BA compound treatment regulates crosstalk between different plant hormone signals, mainly by affecting the expression of related genes in the IAA, CTK, BR, JA and SA signaling pathways. In addition, the phenylpropanoid biosynthesis pathway may also be the key metabolic pathway of maize mesocotyls in response to exogenous SA and 6-BA signals under deep-sowing conditions. The biosynthesis of secondary metabolites such as lignin plays an important role in regulating the elongation of maize mesocotyls.

## Data Availability

The sequencing data of this study were stored in the SRA database of NCBI, and the accession number was PRJNA1063031.

## Supplementary Materials

The following supporting information can be downloaded at: https://www.mdpi.com/article/10.3390/ijms25116150/s1.
