# Supplementary material for: The Mechanism of Exogenous Salicylic Acid and 6-Benzylaminopurine Regulating the Elongation of Maize Mesocotyl
Source: Int J Mol Sci. 2024 Jun 3;25(11):6150. doi: 10.3390/ijms25116150 (PMC11172663; doi:10.3390/ijms25116150)
Supplement: Supplementary file 1 [file ijms-25-06150-s001.zip › ijms-2979521-supplementary.pdf]

International Journal of *Molecular Sciences*

MDPI

Supplementary Material

The Mechanism of Exogenous Salicylic Acid and 6-Benzylaminopurine Regulating the Elongation of Maize Mesocotyl

Xue Qi[1, 2, 3,] Zelong Zhuang[1, 2, 3,] Xiangzhuo Ji[1,2,3,] Jianwen Bian[1,2,3,] and Yunling peng[1, 2, 3, *]

1 College of Agronomy, Gansu Agricultural University, Lanzhou, China
2 Gansu Provincial Key Laboratory of Aridland Crop Science, Gansu Agricultural University,Lanzhou, China
3 Gansu Key Laboratory of Crop Improvement & Germplasm Enhancement, Gansu Agricultural University, Lanzhou, China
* Correspondence: pengyl@gsau.edu.cn

1. Supplementary Tables and Figures

1.1Supplementary Tables

Table S1 Comprehensive evaluation of the optimal concentration under exogenous SA treatment

	RL	SL	MES	COL	MES+COL	Membership average	Rank
CK	1.00	1.00	0.14	0.21	0.15	0.50	5
DS	0.60	0.57	0.54	0.88	0.62	0.64	2
S1	0.60	0.39	0.50	0.64	0.53	0.53	4
S2	0.63	0.51	1.00	1.00	1.00	0.83	1
S3	0.47	0.55	0.40	0.86	0.50	0.55	3
S4	0.17	0.00	0.15	0.00	0.10	0.08	6
S5	0.00	0.09	0.00	0.05	0.00	0.03	7

Table S2 Comprehensive evaluation of the optimal concentration under exogenous 6-BA treatment

	RL	SL	MES	COL	MES+COL	Membership average	Rank
CK	1.00	1.00	0.53	0.00	0.38	0.58	3
DS	0.76	0.65	1.00	0.70	1.00	0.82	1
B1	0.25	0.34	0.80	1.00	0.92	0.66	2
B2	0.11	0.35	0.42	0.73	0.51	0.42	4
B3	0.09	0.00	0.38	0.77	0.49	0.35	5
B4	0.02	0.03	0.11	0.80	0.27	0.25	6
B5	0.00	0.07	0.00	0.25	0.00	0.06	7

Table S3 Comprehensive evaluation of the optimal concentration under exogenous SA-6-BA combined treatment

	RL	SL	MES	COL	MES+COL	Membership average	Rank
DS	1.00	1.00	0.57	0.05	0.47	0.62	2
S2	0.83	0.91	1.00	0.54	1.00	0.85	1
B3	0.00	0.36	0.00	1.00	0.25	0.32	4
C1	0.03	0.14	0.08	0.18	0.08	0.10	7
C2	0.04	0.00	0.05	0.00	0.00	0.02	8
C3	0.15	0.38	0.16	0.67	0.29	0.33	3
C4	0.06	0.23	0.06	0.87	0.27	0.30	5
C5	0.16	0.19	0.09	0.56	0.21	0.24	6

Table S4 RNA concentration and quality detection of sequencing samples

Sample ID	Conc. (ng/ul)	OD260/280	OD260/230	RIN value	28S/18S
QCK-1	58.1	2.12	1.83	9.5	2.16
QCK-2	50.3	2.06	1.89	9.7	2.26
QCK-3	47.4	1.98	2	10	2.17
QCKS-1	60.7	2.02	1.93	9.5	2.29
QCKS-2	69.5	2.05	1.9	9.7	2.18
QCKS-3	72.6	2.03	1.96	10	1.86
QCKB-1	58.81	2.09	2.09	9.3	1.55
QCKB-2	69.61	2.1	1.9	9	1.93
QCKB-3	89.49	2.12	2.21	9.3	1.75
QCKC-1	34.37	2.14	2	8.9	1.88
QCKC-2	45.48	2.14	2.15	8.2	2.1
QCKC-3	69.19	2.04	2.11	9.4	1.96

Sample ID	Conc. (ng/ul)	OD260/280	OD260/230	RIN value	28S/18S
QDS-1	44.58	2.2	1.64	9.3	2.12
QDS-2	54.54	2.11	1.73	9.3	2.3
QDS-3	38.93	2.17	1.82	8.8	1.93
QDSS-1	50.53	2.14	1.92	8.6	2.57
QDSS-2	54.18	2.23	2.05	9.1	2.18
QDSS-3	49.46	2.04	1.7	9.4	1.98
QDSB-1	67.69	2.06	1.68	9.4	2.14
QDSB-2	54.65	2.12	1.84	9.7	1.89
QDSB-3	69.03	2.14	2.11	9.3	1.86
QDSC-1	47.38	2.17	1.76	9.2	2
QDSC-2	49.09	2.14	2.07	9.3	1.92
QDSC-3	28.5	2.25	1.33	9.7	1.88
ZCK-1	26.35	2.26	1.42	8.3	2.09
ZCK-2	42.2	2.14	2.03	7.7	2.44
ZCK-3	63.5	2.14	1.97	9	1.98
ZCKS-1	63.2	2.19	1.8	8.5	1.82
ZCKS-2	74.38	2.18	1.79	8.2	2.33
ZCKS-3	61.88	2.08	1.97	9.3	1.94
ZCKB-1	113.52	2.13	2.08	9.4	2.36
ZCKB-2	296.74	2.05	2.35	8.8	1.83
ZCKB-3	117.91	2.06	2.08	9.4	1.76
ZCKC-1	72.27	2.05	2.12	8.4	2.22
ZCKC-2	109.17	2.04	2.1	9.2	1.8
ZCKC-3	114.35	2.05	2.23	9.2	2.02
ZDS-1	49.6	2.04	1.72	7.8	2.5
ZDS-2	50.95	2.18	1.86	9.4	1.96
ZDS-3	42.04	1.98	1.9	8.6	2.47
ZDSS-1	68.32	2.03	1.73	8.8	2.51
ZDSS-2	63.57	2	1.73	9.1	2.14
ZDSS-3	18.7	2.06	1.49	8.1	2.3
ZDSB-1	63.7	2.04	2.28	7.4	2.23
ZDSB-2	98.12	2.02	2.21	8.3	2.47
ZDSB-3	48.59	2.1	2.16	8	2.42
ZDSC-1	55.01	2.06	1.79	9.2	2.25
ZDSC-2	66.2	1.99	1.82	8.7	2.43
ZDSC-3	26.19	2.06	1.37	8.7	2.39

Table S5 Quality control of sequencing data

Sample ID	Clean reads	Clean bases	GC content (%)	Q30 (%)
QCK-1	23640358	7.08G	52.59	92.5
QCK-2	23909779	7.15G	52.91	93.36
QCK-3	23936193	7.17G	52.64	93.42
QCKB-1	24207988	7.25G	53.9	92.98
QCKB-2	25062184	7.51G	53.88	92.91
QCKB-3	23176787	6.94G	53.9	93.16
QCKC-1	21323585	6.39G	53.95	92.88
QCKC-2	21695100	6.50G	53.56	93.06
QCKC-3	23556305	7.06G	53.35	92.75

Sample ID	Clean reads	Clean bases	GC content (%)	Q30 (%)
QCKS-1	24884394	7.45G	53.02	92.55
QCKS-2	24210343	7.25G	52.71	93.12
QCKS-3	20174908	6.04G	52.88	93.19
QDS-1	21644990	6.48G	53.37	93.08
QDS-2	21732906	6.51G	52.96	92.84
QDS-3	22450984	6.72G	52.81	93.22
QDSB-1	24112116	7.22G	53.4	93.67
QDSB-2	26866404	8.05G	54.29	93.08
QDSB-3	21396241	6.41G	53.97	92.59
QDSC-1	23919666	7.16G	53.2	92.79
QDSC-2	20111163	6.02G	53.51	92.76
QDSC-3	21802359	6.53G	52.96	92.44
QDSS-1	20618421	6.18G	53.69	92.99
QDSS-2	23438980	7.02G	53.06	93.36
QDSS-3	21244490	6.36G	53.07	93.23
ZCK-1	21912739	6.56G	50.94	92.83
ZCK-2	21843505	6.53G	51.54	93.04
ZCK-3	21529400	6.44G	50.57	93.44
ZCKB-1	22272392	6.67G	51.66	92.95
ZCKB-2	21557345	6.46G	53.64	92.26
ZCKB-3	22698346	6.80G	53.01	92.95
ZCKC-1	21759084	6.51G	52.4	93.11
ZCKC-2	23960221	7.17G	51.85	93.15
ZCKC-3	20542476	6.15G	53.17	93.24
ZCKS-1	20814416	6.23G	52.83	93.22
ZCKS-2	21952701	6.57G	51.87	93.36
ZCKS-3	21514508	6.44G	51.58	92.67
ZDS-1	21759562	6.52G	51.51	93.03
ZDS-2	21113961	6.32G	50.79	93.05
ZDS-3	21690251	6.50G	50.53	93.44
ZDSB-1	22160816	6.64G	52.4	92.81
ZDSB-2	22223268	6.66G	52.67	93.42
ZDSB-3	22607899	6.77G	53.25	92.9
ZDSC-1	21495457	6.44G	51.27	93.04
ZDSC-2	23441129	7.02G	50.69	93.45
ZDSC-3	21658553	6.49G	51.24	92.92
ZDSS-1	21192794	6.35G	51.94	92.47
ZDSS-2	22958536	6.88G	51.69	93.24
ZDSS-3	21921311	6.56G	51.31	93.89

Table S6 Reference genome alignments

Sample	Total Reads	Mapped Reads	Uniq Mapped Reads	Multiple Map Reads
QCK-1	47,280,716	41,455,378 (87.68%)	40,345,559 (85.33%)	1,109,819 (2.35%)
QCK-2	47,819,558	42,093,531 (88.03%)	40,902,799 (85.54%)	1,190,732 (2.49%)

Sample	Total Reads	Mapped Reads	Uniq Mapped Reads	Multiple Map Reads
QCK-3	47,872,386	42,465,594 (88.71%)	41,276,252 (86.22%)	1,189,342 (2.48%)
QCKB-1	48,415,976	40,872,273 (84.42%)	39,631,752 (81.86%)	1,240,521 (2.56%)
QCKB-2	50,124,368	41,107,700 (82.01%)	39,822,967 (79.45%)	1,284,733 (2.56%)
QCKB-3	46,353,574	40,833,524 (88.09%)	39,746,412 (85.75%)	1,087,112 (2.35%)
QCKC-1	42,647,170	35,334,973 (82.85%)	34,202,367 (80.20%)	1,132,606 (2.66%)
QCKC-2	43,390,200	35,118,781 (80.94%)	33,956,694 (78.26%)	1,162,087 (2.68%)
QCKC-3	47,112,610	39,546,935 (83.94%)	38,266,770 (81.22%)	1,280,165 (2.72%)
QCKS-1	49,768,788	43,575,530 (87.56%)	42,349,800 (85.09%)	1,225,730 (2.46%)
QCKS-2	48,420,686	42,801,096 (88.39%)	41,635,530 (85.99%)	1,165,566 (2.41%)
QCKS-3	40,349,816	35,956,088 (89.11%)	35,012,503 (86.77%)	943,585 (2.34%)
QDS-1	43,289,980	38,146,985 (88.12%)	37,145,652 (85.81%)	1,001,333 (2.31%)
QDS-2	43,465,812	38,234,725 (87.97%)	37,222,545 (85.64%)	1,012,180 (2.33%)
QDS-3	44,901,968	38,979,204 (86.81%)	37,834,099 (84.26%)	1,145,105 (2.55%)
QDSB-1	48,224,232	39,672,163 (82.27%)	38,330,882 (79.48%)	1,341,281 (2.78%)
QDSB-2	53,732,808	44,421,306 (82.67%)	42,897,384 (79.83%)	1,523,922 (2.84%)
QDSB-3	42,792,482	35,687,603 (83.40%)	34,460,001 (80.53%)	1,227,602 (2.87%)
QDSC-1	47,839,332	38,651,769 (80.79%)	37,355,592 (78.09%)	1,296,177 (2.71%)
QDSC-2	40,222,326	32,671,535 (81.23%)	31,539,152 (78.41%)	1,132,383 (2.82%)
QDSC-3	43,604,718	34,918,487 (80.08%)	33,606,940 (77.07%)	1,311,547 (3.01%)
QDSS-1	41,236,842	34,909,753 (84.66%)	33,888,653 (82.18%)	1,021,100 (2.48%)
QDSS-2	46,877,960	41,621,974 (88.79%)	40,526,206 (86.45%)	1,095,768 (2.34%)
QDSS-3	42,488,980	37,393,240 (88.01%)	36,378,717 (85.62%)	1,014,523 (2.39%)
ZCK-1	43,825,478	36,753,825 (83.86%)	35,637,445 (81.32%)	1,116,380 (2.55%)
ZCK-2	43,687,010	36,149,923 (82.75%)	35,153,171 (80.47%)	996,752 (2.28%)
ZCK-3	43,058,800	36,257,416 (84.20%)	35,190,882 (81.73%)	1,066,534 (2.48%)
ZCKB-1	44,544,784	37,993,287 (85.29%)	36,840,959 (82.71%)	1,152,328 (2.59%)
ZCKB-2	43,114,690	34,471,597 (79.95%)	33,300,994 (77.24%)	1,170,603 (2.72%)
ZCKB-3	45,396,692	38,712,790 (85.28%)	37,638,578 (82.91%)	1,074,212 (2.37%)
ZCKC-1	43,518,168	37,469,588 (86.10%)	36,209,835 (83.21%)	1,259,753 (2.89%)
ZCKC-2	47,920,442	40,917,134 (85.39%)	39,758,054 (82.97%)	1,159,080 (2.42%)
ZCKC-3	41,084,952	35,272,759 (85.85%)	34,235,576 (83.33%)	1,037,183 (2.52%)
ZCKS-1	41,628,832	35,263,896 (84.71%)	34,351,953 (82.52%)	911,943 (2.19%)
ZCKS-2	43,905,402	36,622,862 (83.41%)	35,587,138 (81.05%)	1,035,724 (2.36%)
ZCKS-3	43,029,016	36,641,984 (85.16%)	35,581,190 (82.69%)	1,060,794 (2.47%)
ZDS-1	43,519,124	36,870,595 (84.72%)	35,838,844 (82.35%)	1,031,751 (2.37%)
ZDS-2	42,227,922	35,450,282 (83.95%)	34,406,887 (81.48%)	1,043,395 (2.47%)
ZDS-3	43,380,502	36,483,336 (84.10%)	35,424,826 (81.66%)	1,058,510 (2.44%)
ZDSB-1	44,321,632	37,606,107 (84.85%)	36,595,667 (82.57%)	1,010,440 (2.28%)
ZDSB-2	44,446,536	37,001,141 (83.25%)	35,958,802 (80.90%)	1,042,339 (2.35%)
ZDSB-3	45,215,798	36,420,595 (80.55%)	35,418,579 (78.33%)	1,002,016 (2.22%)
ZDSC-1	42,990,914	36,625,756 (85.19%)	35,624,348 (82.86%)	1,001,408 (2.33%)
ZDSC-2	46,882,258	39,704,138 (84.69%)	38,603,966 (82.34%)	1,100,172 (2.35%)
ZDSC-3	43,317,106	35,729,492 (82.48%)	34,642,607 (79.97%)	1,086,885 (2.51%)
ZDSS-1	42,385,588	32,965,953 (77.78%)	31,800,895 (75.03%)	1,165,058 (2.75%)
ZDSS-2	45,917,072	39,241,559 (85.46%)	38,210,253 (83.22%)	1,031,306 (2.25%)
ZDSS-3	43,842,622	36,804,585 (83.95%)	35,805,164 (81.67%)	999,421 (2.28%)

Table S7 The common DEGs in phenylpropanoid biosynthesis pathway of two inbred lines under SA treatment

Gene ID	GO_annotation	KEGG_annotation
Zm00001eb417240	peroxidase activity; extracellular region; response to oxidative stress; plant-type cell wall; plasmodesma; heme binding; hydrogen peroxide catabolic process; metal ion binding	peroxidase; uncharacterized protein LOC100217011 precursor
Zm00001eb277770	oxidoreductase activity; FAD binding	cinnamyl-alcohol dehydrogenase; inactive tetrahydrocannabinolic acid synthase
Zm00001eb316910	catalytic activity	cinnamoyl-CoA reductase; cinnamoyl-CoA reductase 1-like
Zm00001eb214870	hydrolase activity, hydrolyzing O-glycosyl compounds; carbohydrate metabolic process	beta-glucosidase; uncharacterized protein LOC110431790 isoform X1
Zm00001eb211960	catalytic activity	cinnamoyl-CoA reductase; cinnamoyl-CoA reductase 1-like isoform X1
Zm00001eb160830	aldehyde dehydrogenase (NAD+) activity	coniferyl-aldehyde dehydrogenase; aldh5; aldehyde dehydrogenase 5
Zm00001eb404800	transferase activity, transferring acyl groups other than amino-acyl groups	shikimate O-hydroxycinnamoyltransferase; vinorine synthase
Zm00001eb384900	oxidoreductase activity; FAD binding	cinnamyl-alcohol dehydrogenase; berberine bridge enzyme-like 8
Zm00001eb225230	peroxidase activity; extracellular region; response to oxidative stress; heme binding; hydrogen peroxide catabolic process; metal ion binding	peroxidase; peroxidase 39 precursor
Zm00001eb330550	peroxidase activity; response to oxidative stress; heme binding; hydrogen peroxide catabolic process; metal ion binding	peroxidase; peroxidase 42 precursor
Zm00001eb280790	peroxidase activity; extracellular region; response to oxidative stress; heme binding; hydrogen peroxide catabolic process; metal ion binding	peroxidase; uncharacterized protein LOC100191217 precursor
Zm00001eb083140	peroxidase activity; extracellular region; response to oxidative stress; heme binding; hydrogen peroxide catabolic process; metal ion binding	peroxidase; peroxidase 72

Table S8 The common DEGs in plant hormone signal transduction pathway of two inbred lines under 6-BA treatment

Gene ID	GO_annotation	KEGG_annotation
Zm00001eb124840	protein kinase activity; ATP binding; integral component of membrane; polysaccharide binding	brassinosteroid insensitive 1-associated receptor kinase 1; BRASSINOSTEROID INSENSITIVE 1-associated receptor kinase 1-like isoform X1

Gene ID	GO_annotation	KEGG_annotation
Zm00001eb429330	protein dimerization activity	transcription factor MYC2; transcription factor MYC2-like
Zm00001eb187310	DNA-binding transcription factor activity; nucleus; regulation of transcription, DNA-templated; sequence-specific DNA binding	DELLA protein; DELLA protein DWARF8-like isoform X1
Zm00001eb108330	cytoplasm; acid-amino acid ligase activity	auxin responsive GH3 gene family; GH3; indole-3-acetic acid amido synthetase
Zm00001eb142840	response to auxin	SAUR family protein; auxin-responsive protein SAUR50
Zm00001eb135570	nucleus; regulation of transcription, DNA-templated; auxin-activated signaling pathway	auxin-responsive protein IAA; umc1527; uncharacterized protein LOC100192924
Zm00001eb294390	DNA-binding transcription activator activity, RNA polymerase II-specific; nucleus; protein dimerization activity; regulation of long-day photoperiodism, flowering; regulation of short-day photoperiodism, flowering	phytochrome-interacting factor 4; transcription factor PIF4
Zm00001eb147480	amino acid transmembrane transport; plasma membrane; amino acid transmembrane transporter activity; integral component of membrane	auxin influx carrier (AUX1 LAX family); auxin transporter-like protein 1
Zm00001eb030400	DNA binding; nucleus	two-component response regulator ARR-B family; two-component response regulator ARR12
Zm00001eb233310	DNA binding; nucleus	two-component response regulator ARR-B family; two-component response regulator ARR18-like
Zm00001eb314780	protein serine/threonine kinase activity; ATP binding; integral component of membrane; carbohydrate binding	brassinosteroid insensitive 1-associated receptor kinase 1; BRASSINOSTEROID INSENSITIVE 1-associated receptor kinase 1-like
Zm00001eb330760	protein kinase activity; ATP binding; integral component of membrane	protein brassinosteroid insensitive 1; Os01g0718300, D61, DWARF_61; Similar to Systemin receptor SR160 precursor (Brassinosteroid LRR receptor kinase)
Zm00001eb219160	protein serine/threonine kinase activity; ATP binding; nucleus; cytoplasm; protein phosphorylation; intracellular signal transduction	serine/threonine-protein kinase SRK2; SnRK2.10
Zm00001eb052590	response to auxin	SAUR family protein; auxin-responsive protein SAUR40
Zm00001eb351150	nucleus; mRNA transcription; response to light stimulus	mitogen-activated protein kinase kinase 4/5; mitogen-activated protein kinase kinase 5-like
Zm00001eb084980	nucleus; response to wounding; regulation of defense response; regulation	jasmonate ZIM domain-containing protein; putative tify domain/CCT motif transcription factor family protein

Gene ID	GO_annotation	KEGG_annotation
	of jasmonic acid mediated signaling pathway	
Zm00001eb059460	nucleus; protein dimerization activity	phytochrome-interacting factor 4; transcription factor PIF5 isoform X1
Zm00001eb144290	phosphorelay signal transduction system; DNA binding; DNA-binding transcription factor activity; Cellular Component: nucleus	two-component response regulator ARR-B family; two-component response regulator ARR11
Zm00001eb134820	plasma membrane; integral component of membrane; kinase activity	protein brassinosteroid insensitive 1; systemin receptor SR160
Zm00001eb023010	DNA-binding transcription factor activity; nucleus; regulation of transcription, DNA-templated; sequence-specific DNA binding	DELLA protein
Zm00001eb292830	DNA binding; nucleus; regulation of transcription, DNA-templated; auxin-activated signaling pathway	auxin response factor; ARF23; auxin response factor 23
Zm00001eb273370	protein serine/threonine kinase activity; ATP binding; integral component of membrane	protein brassinosteroid insensitive 1; systemin receptor SR160
Zm00001eb326660	protein serine/threonine kinase activity; ATP binding; plasma membrane; integral component of membrane	protein brassinosteroid insensitive 1; systemin receptor SR160-like
Zm00001eb433460	DNA binding; nucleus; regulation of transcription, DNA-templated; auxin-activated signaling pathway	auxin response factor; auxin response factor 5-like
Zm00001eb177520	protein serine/threonine kinase activity; ATP binding; plasma membrane; hormone-mediated signaling pathway; integral component of membrane	protein brassinosteroid insensitive 1; systemin receptor SR160
Zm00001eb227200	DNA binding; nucleus	two-component response regulator ARR-B family; two-component response regulator ARR18-like
Zm00001eb133000	DNA binding; nucleus; regulation of transcription, DNA-templated; auxin-activated signaling pathway	auxin response factor; auxin response factor 1
Zm00001eb380440	kinase activity	brassinosteroid insensitive 1-associated receptor kinase 1; BRASSINOSTEROID INSENSITIVE 1-associated receptor kinase 1
Zm00001eb277790	protein serine/threonine kinase activity; ATP binding; cytoplasm; cytosol; signal transduction	protein brassinosteroid insensitive 2; putative glycogen synthase kinase family protein
Zm00001eb199570	protein serine/threonine phosphatase activity; magnesium-dependent protein serine/threonine phosphatase activity	protein phosphatase 2C; probable protein phosphatase 2C 75
Zm00001eb361520	protein serine/threonine kinase activity; ATP binding; plasma membrane;	protein brassinosteroid insensitive 1; brassinosteroid LRR receptor kinase precursor

Gene ID	GO_annotation	KEGG_annotation
	hormone-mediated signaling pathway; integral component of membrane	
Zm00001eb045800	nucleus; mRNA transcription; response to light stimulus	mitogen-activated protein kinase kinase 4/5; mitogen-activated protein kinase kinase 5-like
Zm00001eb070460	protein serine/threonine kinase activity; ATP binding; plasma membrane; protein autophosphorylation	brassinosteroid insensitive 1-associated receptor kinase 1; BRASSINOSTEROID INSENSITIVE 1-associated receptor kinase 1-like isoform X1
Zm00001eb082700	DNA-binding transcription factor activity; nucleus; regulation of transcription, DNA-templated; sequence-specific DNA binding	DELLA protein; Sm_DELLA1; GRAS-family protein
Zm00001eb258220	nucleus; regulation of transcription, DNA-templated; auxin-activated signaling pathway	auxin-responsive protein IAA; IAA9 - auxin-responsive Aux/IAA family member
Zm00001eb232120	DNA binding; nucleus; regulation of transcription, DNA-templated; auxin-activated signaling pathway	auxin response factor; auxin response factor 19
Zm00001eb120960	protein dimerization activity	transcription factor MYC2; transcription factor MYC2
Zm00001eb368530	nucleus; mRNA transcription; response to light stimulus	mitogen-activated protein kinase kinase 4/5; mitogen-activated protein kinase kinase 5-like
Zm00001eb307550	DNA binding; DNA-binding transcription factor activity; nucleus	ethylene-responsive transcription factor 1; putative AP2/EREBP transcription factor superfamily protein
Zm00001eb365420	phosphorelay signal transduction system; DNA binding; DNA-binding transcription factor activity; nucleus	two-component response regulator ARR-B family; two-component response regulator ORR26
Zm00001eb015890	protein kinase activity; ATP binding; integral component of membrane	protein brassinosteroid insensitive 1; protein BRASSINOSTEROID INSENSITIVE 1
Zm00001eb164480	DNA-binding transcription factor activity; nucleus; regulation of transcription, DNA-templated; sequence-specific DNA binding	DELLA protein; DELLA protein GAI
Zm00001eb266160	protein serine/threonine kinase activity; ATP binding; integral component of membrane; recognition of pollen	brassinosteroid insensitive 1-associated receptor kinase 1; BRASSINOSTEROID INSENSITIVE 1-associated receptor kinase 1-like
Zm00001eb309030	protein serine/threonine kinase activity; ATP binding; plasma membrane; hormone-mediated signaling pathway; integral component of membrane	protein brassinosteroid insensitive 1; systemin receptor SR160-like
Zm00001eb190410	phosphorelay sensor kinase activity; nucleoside metabolic process	arabidopsis histidine kinase 2/3/4 (cytokinin receptor); probable histidine kinase 4
Zm00001eb223590	transcription coregulator activity; intracellular; obsolete cell; nucleus;	jasmonate ZIM domain-containing protein; protein TIFY 11d

Gene ID	GO_annotation	KEGG_annotation
	response to stress; response to wounding; regulation of biosynthetic process; negative regulation of biosynthetic process; negative regulation of metabolic process; regulation of signal transduction; regulation of macromolecule biosynthetic process; transcription regulator activity; negative regulation of RNA biosynthetic process; regulation of nucleic acid-templated transcription; negative regulation of nucleic acid-templated transcription; regulation of jasmonic acid mediated signaling pathway; regulation of RNA biosynthetic process	
Zm00001eb152200	cytoplasm; acid-amino acid ligase activity	auxin responsive GH3 gene family; uncharacterized protein LOC100383126
Zm00001eb037990	protein serine/threonine kinase activity; ATP binding; integral component of membrane; recognition of pollen	brassinosteroid insensitive 1-associated receptor kinase 1; BRASSINOSTEROID INSENSITIVE 1-associated receptor kinase 1
Zm00001eb135550	nucleus; regulation of transcription, DNA-templated; auxin-activated signaling pathway	auxin-responsive protein IAA; IAA30 - auxin-responsive Aux/IAA family member
Zm00001eb282800	phosphorelay sensor kinase activity; endoplasmic reticulum; integral component of membrane; ethylene receptor activity; ethylene binding	ethylene receptor; probable ethylene response sensor 2
Zm00001eb216090	phosphorelay sensor kinase activity; integral component of membrane	arabidopsis histidine kinase 2/3/4 (cytokinin receptor); HK1b2; histidine kinase
Zm00001eb191370	nucleus; regulation of transcription, DNA-templated; auxin-activated signaling pathway	auxin-responsive protein IAA; AUX27; hypothetical protein
Zm00001eb368950	protein kinase activity; ATP binding; integral component of membrane	protein brassinosteroid insensitive 1
Zm00001eb284310	nucleus; regulation of transcription, DNA-templated; Biological Process: auxin-activated signaling pathway	auxin-responsive protein IAA; IAA17-auxin-responsive Aux/IAA family member
Zm00001eb408800	DNA binding; nucleus; regulation of transcription, DNA-templated; auxin-activated signaling pathway	auxin response factor; auxin response factor 1
Zm00001eb098220	protein serine/threonine phosphatase activity; magnesium-dependent protein serine/threonine phosphatase activity; nucleus; cytosol	protein phosphatase 2C; probable protein phosphatase 2C 68 isoform 2
Zm00001eb102510	response to auxin	SAUR family protein; auxin-responsive protein SAUR72
Zm00001eb332400	protein dimerization activity	phytochrome-interacting factor 3; transcription factor APG-like isoform X1

Gene ID	GO_annotation	KEGG_annotation
Zm00001eb239210	protein kinase activity; ATP binding; integral component of membrane	brassinosteroid insensitive 1-associated receptor kinase 1; BRASSINOSTEROID INSENSITIVE 1-associated receptor kinase 1-like isoform X1
Zm00001eb164530	DNA-binding transcription factor activity; nucleus; regulation of transcription, DNA-templated; sequence-specific DNA binding	DELLA protein; DELLA protein GAI-like
Zm00001eb066640	DNA binding; nucleus; regulation of transcription, DNA-templated; auxin-activated signaling pathway	auxin response factor; auxin response factor 19 isoform X3
Zm00001eb341580	extracellular region	pathogenesis-related protein 1; TIDP2793; pathogenesis-related protein PRMS precursor
Zm00001eb360810	protein serine/threonine kinase activity; ATP binding; plasma membrane; hormone-mediated signaling pathway; integral component of membrane	protein brassinosteroid insensitive 1; brassinosteroid LRR receptor kinase BRI1
Zm00001eb320950	DNA-binding transcription factor activity; protein binding; regulation of transcription, DNA-templated; regulation of biosynthetic process; regulation of gene expression; regulation of macromolecule biosynthetic process; regulation of nucleobase-containing compound metabolic process	DELLA protein; uncharacterized protein LOC18106830
Zm00001eb149120	nucleus; mRNA transcription; response to light stimulus	mitogen-activated protein kinase kinase 4/5; mitogen-activated protein kinase kinase 5-like
Zm00001eb243440	protein serine/threonine kinase activity; ATP binding; nucleus; cytoplasm; protein phosphorylation; intracellular signal transduction	serine/threonine-protein kinase SRK2; uncharacterized protein LOC542190
Zm00001eb350370	nucleus; regulation of transcription, DNA-templated; auxin-activated signaling pathway	auxin-responsive protein IAA; umc1460; uncharacterized protein LOC100274569 isoform 1
Zm00001eb326420	cytoplasm; acid-amino acid ligase activity	auxin responsive GH3 gene family; indole-3-acetic acid-amido synthetase GH3.8
Zm00001eb389770	DNA binding; nucleus	two-component response regulator ARR-B family; hypothetical protein
Zm00001eb324600	endodermal cell fate specification; DNA-binding transcription factor activity; nucleus; regulation of transcription, DNA-templated; sequence-specific DNA binding; leaf development; bundle sheath cell fate specification	DELLA protein; DELLA protein GAI-like

Table S9 Five candidate genes screened by WGCNA

Gene ID	GO_annotation	KEGG_annotation
Zm00001eb194640	auxin-activated signaling pathway; integral component of membrane; transmembrane transport	auxin efflux carrier family protein; auxin efflux carrier component 1a
Zm00001eb159150	uroporphyrin-III C-methyltransferase activity; siroheme biosynthetic process; methylation; oxidation-reduction process	uroporphyrin-III C-methyltransferase; sum1; siroheme uroporphyrinogen methyltransferase 1
Zm00001eb286960	NA	BRCA1-A complex subunit BRE; BRISC and BRCA1-A complex member 2
Zm00001eb428310	ATP binding; integral component of membrane; ATPase activity; ATPase-coupled transmembrane transporter activity	ATP-binding cassette, subfamily C (CFTR/MRP), member 1; putative ABC transporter C family member 15
Zm00001eb149840	NA	NA

Table S10 The common DEGs of the two inbred lines under SA treatment

Gene ID	GO_annotation	KEGG_annotation
NewGene_10159	NA	NA
Zm00001eb020740	nitrogen compound metabolic process; multicellular organism development; biological process; metabolic process; plant organ development; plant organ morphogenesis	NA
Zm00001eb048770	nucleus; response to wounding; regulation of defense response; regulation of jasmonic acid mediated signaling pathway	jasmonate ZIM domain-containing protein; ZIM motif family protein
Zm00001eb140600	calcium ion binding	calcium-binding protein CML; putative calcium-binding protein CML19
Zm00001eb214250	NA	NA
Zm00001eb324520	NA	NA
Zm00001eb347180	calcium ion binding	calcium-binding protein CML; polcalcin Jun o 2
Zm00001eb384060	NA	NA
Zm00001eb399590	oxygen carrier activity; oxygen binding; heme binding; metal ion binding	ariadne-1; probable E3 ubiquitin-protein ligase ARI2 isoform X1
Zm00001eb404780	iron ion binding; membrane; integral component of membrane; oxidoreductase activity, acting on paired donors, with incorporation or reduction of molecular oxygen, NAD(P)H as one donor, and incorporation of one atom of oxygen; heme binding	cytochrome P450 family 89 subfamily A; cytochrome P450 89A2
Zm00001eb404800	transferase activity, transferring acyl groups other than amino-acyl groups	shikimate O-hydroxycinnamoyltransferase; vinorine synthase
Zm00001eb413930	DNA binding; DNA-binding transcription factor activity; nucleus	EREBP-like factor; ethylene-responsive transcription factor ERF098

Table S11 The common DEGs in the phenylpropanoid biosynthesis pathway of the two inbred lines under 6-BA treatment

Gene ID	GO_annotation	KEGG_annotation
Zm00001eb417340	peroxidase activity; response to oxidative stress; heme binding; hydrogen peroxide catabolic process; metal ion binding	peroxidase; LOW QUALITY PROTEIN: peroxidase 5
Zm00001eb308860	ligase activity	4-coumarate--CoA ligase; 4-coumarate--CoA ligase-like 7
Zm00001eb291860	peroxidase activity; extracellular region; response to oxidative stress; plant-type cell wall; plasmodesma; heme binding; hydrogen peroxide catabolic process; metal ion binding	peroxidase; umc2389; uncharacterized protein LOC100284675 precursor
Zm00001eb006430	transferase activity, transferring acyl groups other than amino-acyl groups	shikimate O-hydroxycinnamoyltransferase; LOC109746960; tryptamine benzoyltransferase 1-like
Zm00001eb282410	peroxidase activity; extracellular region; response to oxidative stress; plant-type cell wall	peroxidase; peroxidase 5
Zm00001eb130940	peroxidase activity; extracellular region; response to oxidative stress; plant-type cell wall; plasmodesma	peroxidase; pox1; peroxidase 1
Zm00001eb195210	peroxidase activity; extracellular region; response to oxidative stress; heme binding; hydrogen peroxide catabolic process; metal ion binding	peroxidase; peroxidase 52 precursor
Zm00001eb054230	carbohydrate metabolic process; beta-glucosidase activity	beta-glucosidase; uncharacterized protein LOC100274288 precursor
Zm00001eb348950	peroxidase activity; extracellular region; response to oxidative stress; heme binding; hydrogen peroxide catabolic process; metal ion binding	peroxidase; peroxidase 2
Zm00001eb022050	peroxidase activity; extracellular region; response to oxidative stress; plant-type cell wall; plasmodesma; heme binding; hydrogen peroxide catabolic process; metal ion binding	peroxidase; umc1244; uncharacterized protein LOC100216632 precursor
Zm00001eb214870	hydrolase activity, hydrolyzing O-glycosyl compounds; Biological Process: carbohydrate metabolic process	beta-glucosidase; uncharacterized protein LOC110431790 isoform X1
Zm00001eb205530	ammonia-lyase activity	phenylalanine ammonia-lyase; phenylalanine ammonia-lyase
Zm00001eb211960	catalytic activity	cinnamoyl-CoA reductase; cinnamoyl-CoA reductase 1-like isoform X1
Zm00001eb325930	peroxidase activity; response to oxidative stress; heme binding; metal ion binding	peroxidase; peroxidase 70
Zm00001eb387920	oxidoreductase activity; oxidoreductase activity, acting on the CH-OH group of donors, NAD or NADP as acceptor	cinnamoyl-CoA reductase; CCR2; cinnamoyl-CoA reductase
Zm00001eb238800	peroxidase activity; extracellular region; Biological Process: response to oxidative	peroxidase; uncharacterized protein LOC107546759 precursor

Gene ID	GO_annotation	KEGG_annotation
	stress; heme binding; hydrogen peroxide catabolic process; metal ion binding	
Zm00001eb047120	peroxidase activity; extracellular region; response to oxidative stress; heme binding; hydrogen peroxide catabolic process; metal ion binding	peroxidase; uncharacterized protein LOC100272764 precursor
Zm00001eb076170	peroxidase activity; extracellular region; response to oxidative stress; heme binding; hydrogen peroxide catabolic process; metal ion binding	peroxidase; ap1; anionic peroxidase precursor
Zm00001eb374100	cytosol; zinc ion binding; lignin biosynthetic process; oxidoreductase activity, acting on the CH-OH group of donors, NAD or NADP as acceptor	cinnamyl-alcohol dehydrogenase; putative cinnamyl-alcohol dehydrogenase family protein
Zm00001eb071110	transferase activity, transferring acyl groups other than amino-acyl groups	shikimate O-hydroxycinnamoyltransferase; shikimate O-hydroxycinnamoyltransferase-like
Zm00001eb001560	peroxidase activity; response to oxidative stress; heme binding; hydrogen peroxide catabolic process; metal ion binding	peroxidase; uncharacterized LOC100191905 precursor
Zm00001eb285030	monooxygenase activity; iron ion binding; oxidoreductase activity, acting on paired donors, with incorporation or reduction of molecular oxygen; heme binding	trans-cinnamate 4-monooxygenase; putative cytochrome P450 superfamily protein
Zm00001eb131000	peroxidase activity; extracellular region; response to oxidative stress; plant-type cell wall; plasmodesma; heme binding; hydrogen peroxide catabolic process; metal ion binding	peroxidase; uncharacterized protein LOC100273283
Zm00001eb389420	CoA-ligase activity	4-coumarate--CoA ligase; probable 4-coumarate--CoA ligase 4
Zm00001eb357540	aldehyde dehydrogenase (NAD+) activity	coniferyl-aldehyde dehydrogenase; aldehyde dehydrogenase family 2 member C4
Zm00001eb384900	oxidoreductase activity; FAD binding	cinnamyl-alcohol dehydrogenase; berberine bridge enzyme-like 8
Zm00001eb270810	transferase activity, transferring acyl groups other than amino-acyl groups	shikimate O-hydroxycinnamoyltransferase; shikimate O-hydroxycinnamoyltransferase-like
Zm00001eb077220	cytoplasm; L-phenylalanine catabolic process; cinnamic acid biosynthetic process; ammonia-lyase activity; phenylalanine ammonia-lyase activity	phenylalanine ammonia-lyase; phenylalanine ammonia-lyase
Zm00001eb384110	hydrolase activity, hydrolyzing O-glycosyl compounds; carbohydrate metabolic process	beta-glucosidase; uncharacterized protein LOC100501315 precursor
Zm00001eb226370	peroxidase activity; extracellular region; response to oxidative stress; integral component of membrane; heme binding;	peroxidase; peroxidase 45 precursor

Gene ID	GO_annotation	KEGG_annotation
Zm00001eb140320	hydrogen peroxide catabolic process; metal ion binding peroxidase activity; extracellular region; response to oxidative stress; plant-type cell wall; plasmodesma; heme binding; hydrogen peroxide catabolic process; metal ion binding	peroxidase; TIDP3705; uncharacterized protein LOC100192761 precursor
Zm00001eb006410	transferase activity, transferring acyl groups other than amino-acyl groups	shikimate O-hydroxycinnamoyltransferase; LOC109746960; tryptamine benzoyltransferase 1-like
Zm00001eb307060	oxidoreductase activity; oxidoreductase activity, acting on the CH-OH group of donors, NAD or NADP as acceptor	cinnamoyl-CoA reductase; cncr2; cinnamoyl CoA reductase 2
Zm00001eb267480	plasma membrane; hormone-mediated signaling pathway	beta-glucosidase; beta-glucosidase 12
Zm00001eb211970	catalytic activity	cinnamoyl-CoA reductase; cinnamoyl-CoA reductase 1-like
Zm00001eb277710	oxidoreductase activity; FAD binding	cinnamyl-alcohol dehydrogenase; inactive tetrahydrocannabinolic acid synthase
Zm00001eb151890	hydrolase activity, hydrolyzing O-glycosyl compounds; carbohydrate metabolic process	beta-glucosidase; uncharacterized protein LOC8072136 isoform X1
Zm00001eb225230	peroxidase activity; extracellular region; response to oxidative stress; heme binding; hydrogen peroxide catabolic process; metal ion binding	peroxidase; peroxidase 39 precursor
Zm00001eb180060	transferase activity, transferring acyl groups other than amino-acyl groups	shikimate O-hydroxycinnamoyltransferase; pco096884; uncharacterized protein LOC100284063
Zm00001eb365590	CoA-ligase activity	4-coumarate--CoA ligase; 4-coumarate--CoA ligase-like 5
Zm00001eb311610	UDP-glycosyltransferase activity; intracellular membrane-bounded organelle; quercetin 3-O-glucosyltransferase activity; quercetin 7-O-glucosyltransferase activity	scopoletin glucosyltransferase; LOC109746679; anthocyanin 3'-O-beta-glucosyltransferase-like
Zm00001eb330550	peroxidase activity; response to oxidative stress; heme binding; hydrogen peroxide catabolic process; metal ion binding	peroxidase; pco073925; peroxidase 42 precursor
Zm00001eb088800	zinc ion binding; lignin biosynthetic process; oxidoreductase activity, acting on the CH-OH group of donors, NAD or NADP as acceptor; cinnamyl-alcohol dehydrogenase activity	cinnamyl-alcohol dehydrogenase; putative cinnamyl-alcohol dehydrogenase family protein
Zm00001eb187910	ligase activity	4-coumarate--CoA ligase; probable 4-coumarate--CoA ligase 2
Zm00001eb238780	peroxidase activity; extracellular region; response to oxidative stress; heme binding;	peroxidase; uncharacterized protein LOC100279673 precursor

Gene ID	GO_annotation	KEGG_annotation
	hydrogen peroxide catabolic process; metal ion binding	
Zm00001eb417350	peroxidase activity; extracellular region; response to oxidative stress; plant-type cell wall; plasmodesma; heme binding; hydrogen peroxide catabolic process; metal ion binding	peroxidase; uncharacterized protein LOC100273104 precursor
Zm00001eb027900	transferase activity, transferring acyl groups other than amino-acyl groups	shikimate O-hydroxycinnamoyltransferase; LOC109746960; tryptamine benzoyltransferase 1-like
Zm00001eb330540	peroxidase activity; extracellular region; response to oxidative stress; heme binding; hydrogen peroxide catabolic process; metal ion binding	peroxidase; uncharacterized protein LOC100383323 precursor
Zm00001eb417330	peroxidase activity; extracellular region; response to oxidative stress; plant-type cell wall; plasmodesma; heme binding; hydrogen peroxide catabolic process; metal ion binding	peroxidase; peroxidase 5
Zm00001eb247660	cytoplasm; L-phenylalanine catabolic process; cinnamic acid biosynthetic process; ammonia-lyase activity; phenylalanine ammonia-lyase activity	phenylalanine ammonia-lyase; phenylalanine ammonia-lyase
Zm00001eb426520	hydrolase activity, hydrolyzing O-glycosyl compounds; carbohydrate metabolic process	beta-glucosidase; beta-glucosidase precursor
Zm00001eb359110	oxidoreductase activity; oxidoreductase activity, acting on the CH-OH group of donors, NAD or NADP as acceptor	cinnamoyl-CoA reductase; cinnamoyl-CoA reductase 1-like isoform X1
Zm00001eb365690	beta-glucosidase activity; cellulose catabolic process; scopolin beta-glucosidase activity	beta-glucosidase; beta-glucosidase 4
Zm00001eb411380	hydrolase activity, hydrolyzing O-glycosyl compounds; carbohydrate metabolic process	beta-glucosidase; glu1; 4-hydroxy-7-methoxy-3-oxo-3,4-dihydro-2H-1, 4-benzoxazin-2-yl glucoside beta-D-glucosidase 1, chloroplastic precursor
Zm00001eb384940	oxidoreductase activity; FAD binding	cinnamyl-alcohol dehydrogenase; berberine bridge enzyme-like 15
Zm00001eb312060	zinc ion binding; lignin biosynthetic process; oxidoreductase activity, acting on the CH-OH group of donors, NAD or NADP as acceptor; cinnamyl-alcohol dehydrogenase activity	cinnamyl-alcohol dehydrogenase; probable cinnamyl alcohol dehydrogenase 5
Zm00001eb330580	peroxidase activity; extracellular region; response to oxidative stress; heme binding; hydrogen peroxide catabolic process; metal ion binding	peroxidase; cationic peroxidase 1

Gene ID	GO_annotation	KEGG_annotation
Zm00001eb078220	transferase activity, transferring acyl groups other than amino-acyl groups	shikimate O-hydroxycinnamoyltransferase; TIDP2701; uncharacterized protein LOC100279386 isoform X1
Zm00001eb413850	zinc ion binding; lignin biosynthetic process; oxidoreductase activity, acting on the CH-OH group of donors, NAD or NADP as acceptor; cinnamyl-alcohol dehydrogenase activity	cinnamyl-alcohol dehydrogenase; cl4921_1; uncharacterized protein LOC100281336

Table S12 The common DEGs in the phenylpropanoid biosynthesis pathway of the two inbred lines under SA-6-BA treatment

Gene ID	GO_annotation	KEGG_annotation
Zm00001eb214260	peroxidase activity; extracellular region; response to oxidative stress; heme binding; hydrogen peroxide catabolic process; metal ion binding	peroxidase; peroxidase 51 precursor
Zm00001eb006430	transferase activity, transferring acyl groups other than amino-acyl groups	shikimate O-hydroxycinnamoyltransferase; LOC109746960; tryptamine benzoyltransferase 1-like
Zm00001eb130940	peroxidase activity; extracellular region; response to oxidative stress; plant-type cell wall; plasmodesma; heme binding; hydrogen peroxide catabolic process; metal ion binding	peroxidase; pox1; peroxidase 1
Zm00001eb276250	peroxidase activity; extracellular region; response to oxidative stress; heme binding; hydrogen peroxide catabolic process; metal ion binding	peroxidase; uncharacterized protein LOC100383495 precursor
Zm00001eb195210	peroxidase activity; extracellular region; response to oxidative stress; heme binding; hydrogen peroxide catabolic process; metal ion binding	peroxidase; peroxidase 52 precursor
Zm00001eb076180	peroxidase activity; extracellular region; response to oxidative stress; plant-type cell wall; plasmodesma; heme binding; hydrogen peroxide catabolic process; metal ion binding	peroxidase; uncharacterized protein LOC100194034 precursor
Zm00001eb211960	catalytic activity	cinnamoyl-CoA reductase; cinnamoyl-CoA reductase 1-like isoform X1
Zm00001eb278280	transferase activity, transferring acyl groups other than amino-acyl groups	shikimate O-hydroxycinnamoyltransferase; uncharacterized protein LOC100274269
Zm00001eb041120	catalytic activity	cinnamoyl-CoA reductase; cncr1; cinnamoyl CoA reductase 1
Zm00001eb316940	catalytic activity	cinnamoyl-CoA reductase; cinnamoyl-CoA reductase 1-like
Zm00001eb220390	iron ion binding; lignin biosynthetic process; membrane; oxidoreductase activity, acting on paired donors, with	ferulate-5-hydroxylase; putative cytochrome P450 superfamily protein

Gene ID	GO_annotation	KEGG_annotation
	incorporation or reduction of molecular oxygen, NAD(P)H as one donor, and incorporation of one atom of oxygen; heme binding	
Zm00001eb040790	CoA-ligase activity	4-coumarate--CoA ligase; probable 4-coumarate--CoA ligase 5
Zm00001eb076190	peroxidase activity; extracellular region; response to oxidative stress; plant-type cell wall; plasmodesma; heme binding; hydrogen peroxide catabolic process; metal ion binding	peroxidase; uncharacterized protein LOC100279351 precursor
Zm00001eb332990	peroxidase activity; extracellular region; response to oxidative stress; plant-type cell wall; plasmodesma; heme binding; hydrogen peroxide catabolic process; metal ion binding	peroxidase; uncharacterized protein LOC107457598
Zm00001eb178360	CoA-ligase activity	4-coumarate--CoA ligase; probable 4-coumarate--CoA ligase 1
Zm00001eb285030	monooxygenase activity; iron ion binding; oxidoreductase activity, acting on paired donors, with incorporation or reduction of molecular oxygen; heme binding	trans-cinnamate 4-monooxygenase; putative cytochrome P450 superfamily protein
Zm00001eb131000	peroxidase activity; extracellular region; response to oxidative stress; plant-type cell wall; plasmodesma; heme binding	peroxidase; uncharacterized protein LOC100273283
Zm00001eb374630	oxidoreductase activity; oxidoreductase activity, acting on the CH-OH group of donors, NAD or NADP as acceptor	cinnamoyl-CoA reductase; uncharacterized protein LOC100382657
Zm00001eb270810	transferase activity, transferring acyl groups other than amino-acyl groups	shikimate O-hydroxycinnamoyltransferase; shikimate O-hydroxycinnamoyltransferase-like
Zm00001eb140320	peroxidase activity; extracellular region; response to oxidative stress; plant-type cell wall; plasmodesma; heme binding; hydrogen peroxide catabolic process; metal ion binding	peroxidase; TIDP3705; uncharacterized protein LOC100192761 precursor
Zm00001eb006410	transferase activity, transferring acyl groups other than amino-acyl groups	shikimate O-hydroxycinnamoyltransferase; LOC109746960; tryptamine benzoyltransferase 1-like
Zm00001eb277710	oxidoreductase activity; FAD binding	cinnamyl-alcohol dehydrogenase; inactive tetrahydrocannabinolic acid synthase
Zm00001eb329520	hydrolase activity, hydrolyzing O-glycosyl compounds; carbohydrate metabolic process	beta-glucosidase; pco088410; uncharacterized protein LOC100282438 precursor
Zm00001eb150550	iron ion binding; membrane; integral component of membrane; oxidoreductase activity, acting on paired donors, with incorporation or reduction of molecular oxygen,	5-O-(4-coumaroyl)-D-quinate 3'-monooxygenase; putative cytochrome P450 superfamily protein

Gene ID	GO_annotation	KEGG_annotation
	NAD(P)H as one donor, and incorporation of one atom of oxygen; heme binding	
Zm00001eb352700	UDP-glycosyltransferase activity; intracellular membrane-bounded organelle; quercetin 3-O-glucosyltransferase activity; quercetin 7-O-glucosyltransferase activity	scopoletin glucosyltransferase; scopoletin glucosyltransferase-like
Zm00001eb240910	iron ion binding; lignin metabolic process; membrane;	trans-cinnamate 4-monooxygenase
Zm00001eb234730	zinc ion binding; lignin biosynthetic process; oxidoreductase activity, acting on the CH-OH group of donors, NAD or NADP as acceptor; cinnamyl-alcohol dehydrogenase activity	cinnamyl-alcohol dehydrogenase; cad; probable cinnamyl alcohol dehydrogenase
Zm00001eb027900	transferase activity, transferring acyl groups other than amino-acyl groups	shikimate O-hydroxycinnamoyltransferase; LOC109746960; tryptamine benzoyltransferase 1-like

Table S13 RT-qPCR primer sequences

Gene name	Forward Primer（5'→3'）	Reverse Primer（5'→3'）
Actin	TGAAACCTTCGAATGCCCAG	GATTGGAACCGTGTGGCTCA
Zm00001eb140600	GCCTTCCGGATGTACTCCTC	GAGGTCGAACCTGCAGATCA
Zm00001eb404800	CCACGGTCTCTGACTATGGC	CTTGAAGATGCCGTCGGACT
Zm00001eb106310	CTTCCAGCATCTACTACTACCTC	CGTGTTTCCCAGCATTGTGAT
Zm00001eb220050	CGCCGATCAGGGAGATCAAG	TCCTTCTCGGGGCTAACGTC
Zm00001eb326970	TTCATCGACCGGATGAGCG	GGTCCAGCCGGATGAAGTAG
Zm00001eb258220	CATCGGCAGGAAAGTGGACC	CTGATTTCCTTGTCGTCTTGGC
Zm00001eb363180	TTGCGACAAGCGTGCGA	TTGTAGAGCTGCACCACGTC
Zm00001eb371720	CTCGACCACACCTGGAAGTC	AGAACACGGATGCGGACAAT

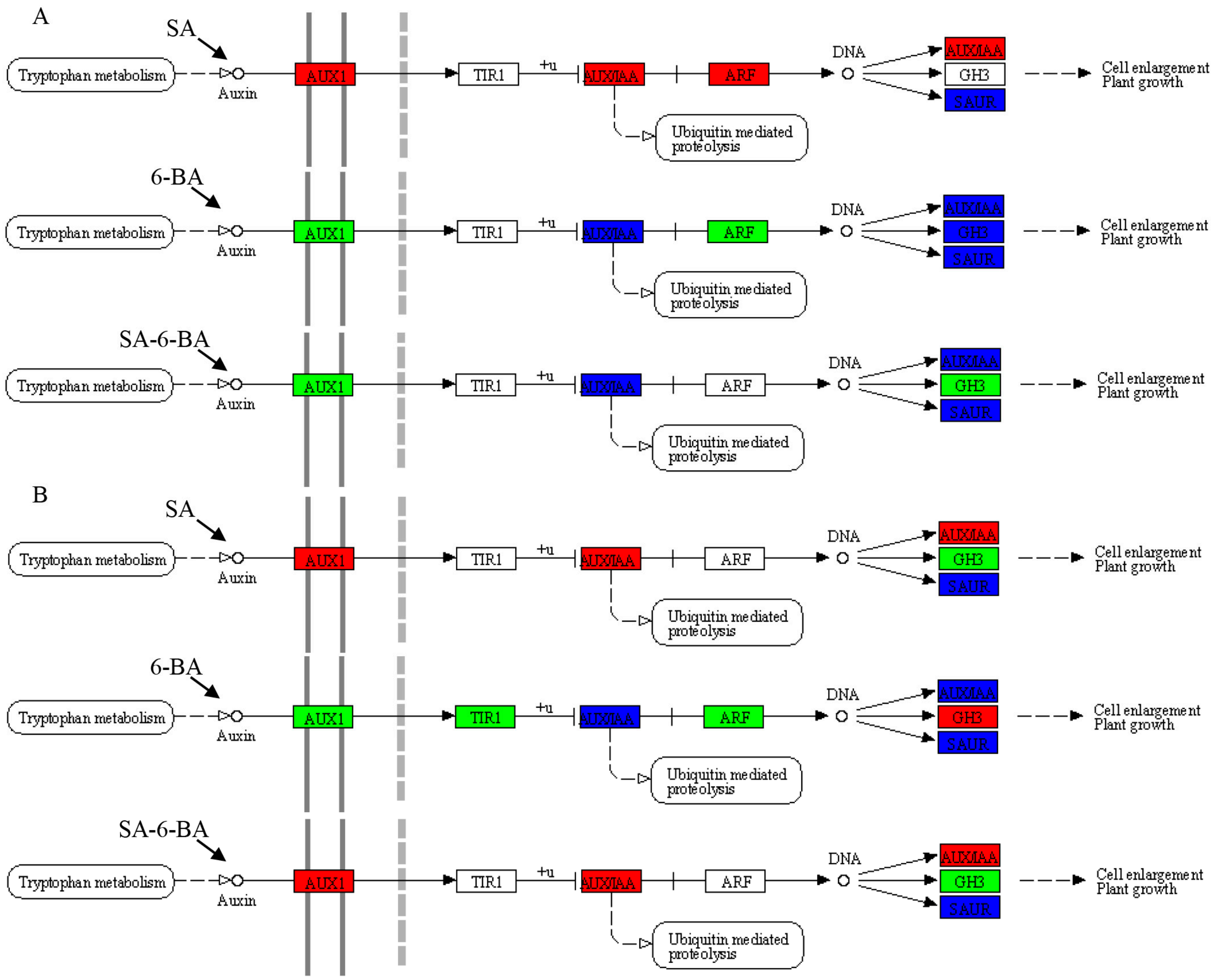

Figure S1. Auxin signaling pathway regulating mesocotyl length by exogenous hormones. (A) is inbred line Qi319; (B) is inbred line Zi330. Red represents up-regulation, green represents down-regulation, and blue represents both up-regulation and down-regulation. The same below.

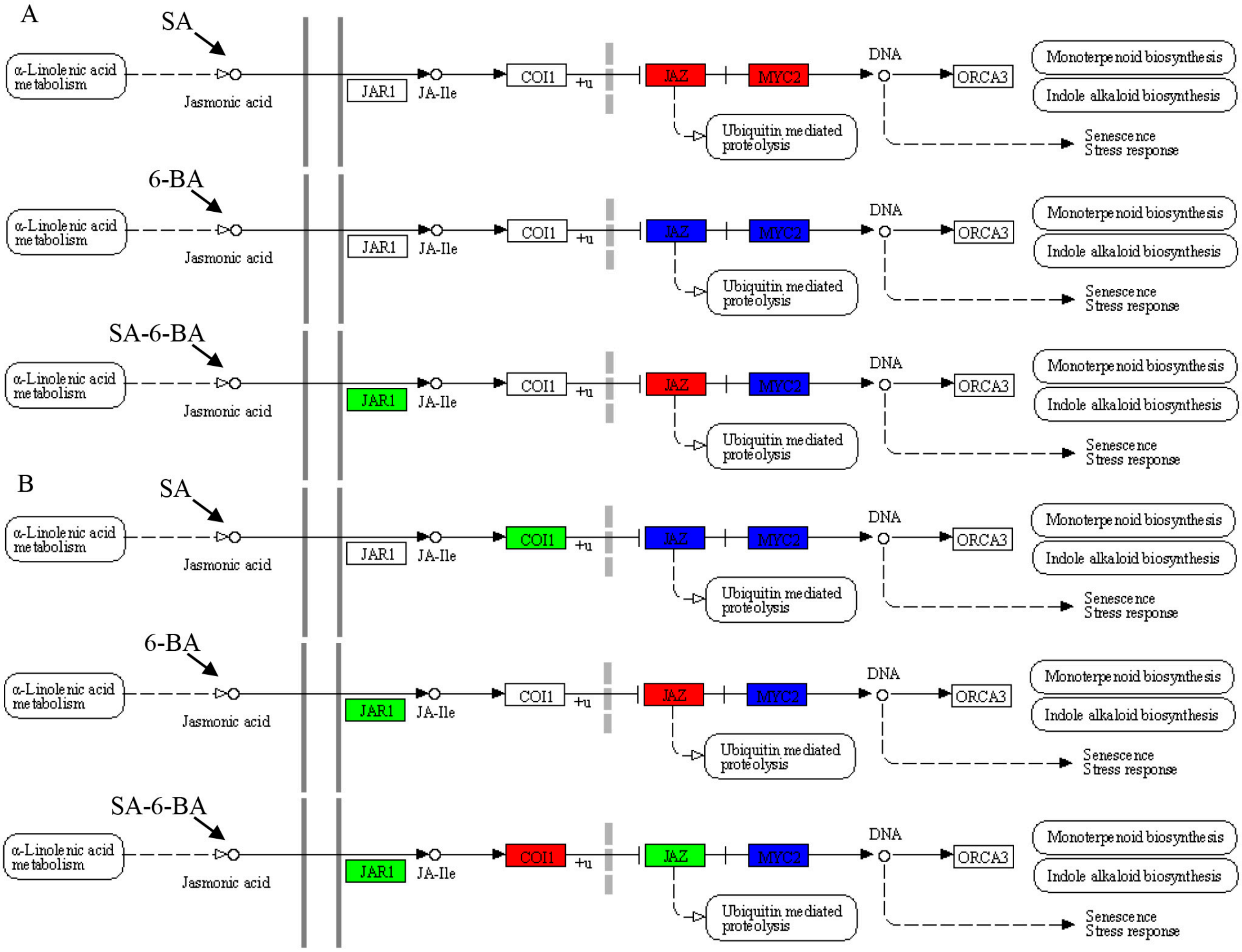

Figure S2. Jasmonic acid signaling pathway regulated by exogenous hormones in mesocotyl length.

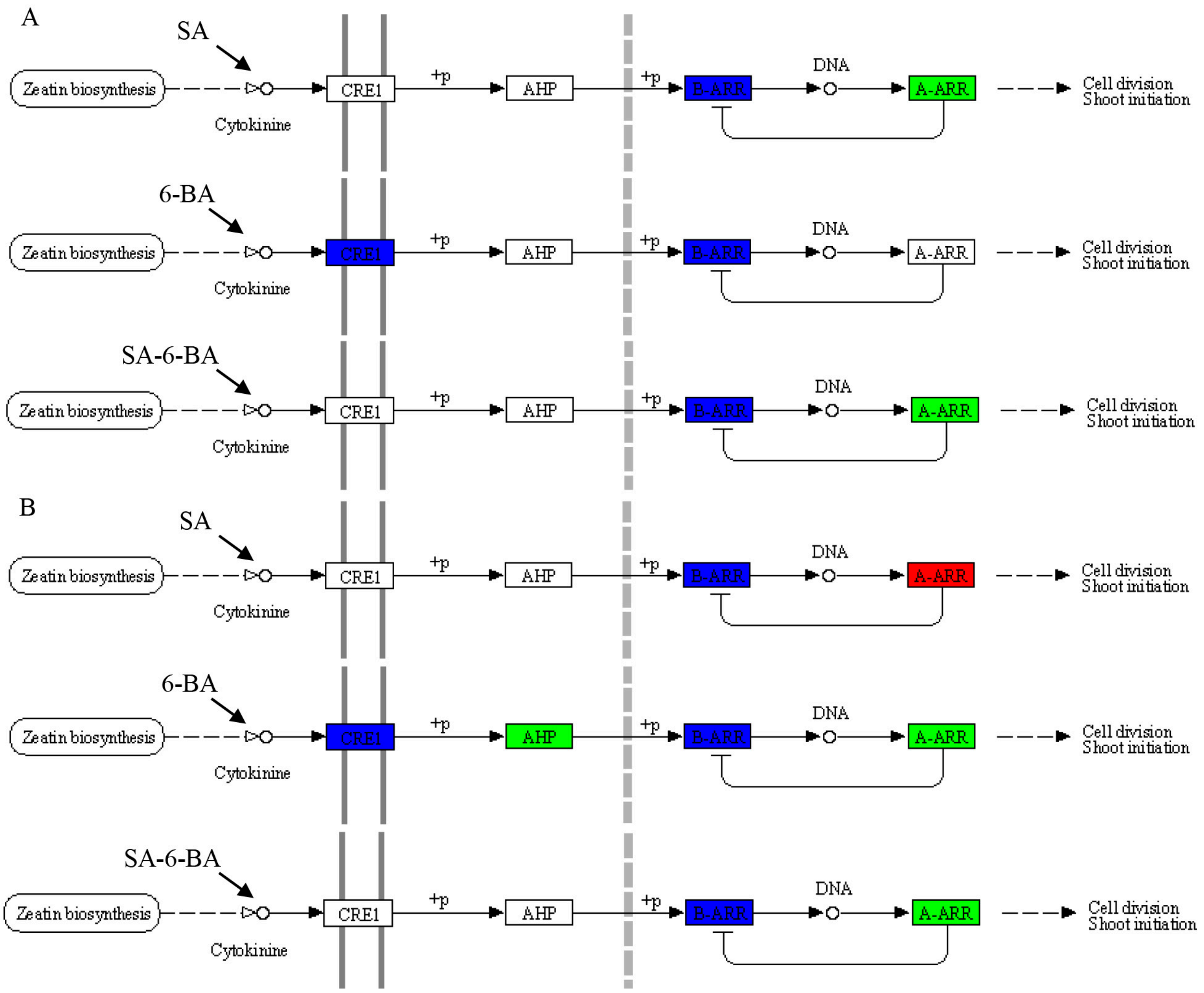

Figure S3. Cytokinin signaling pathway of exogenous hormones regulating mesocotyl length.

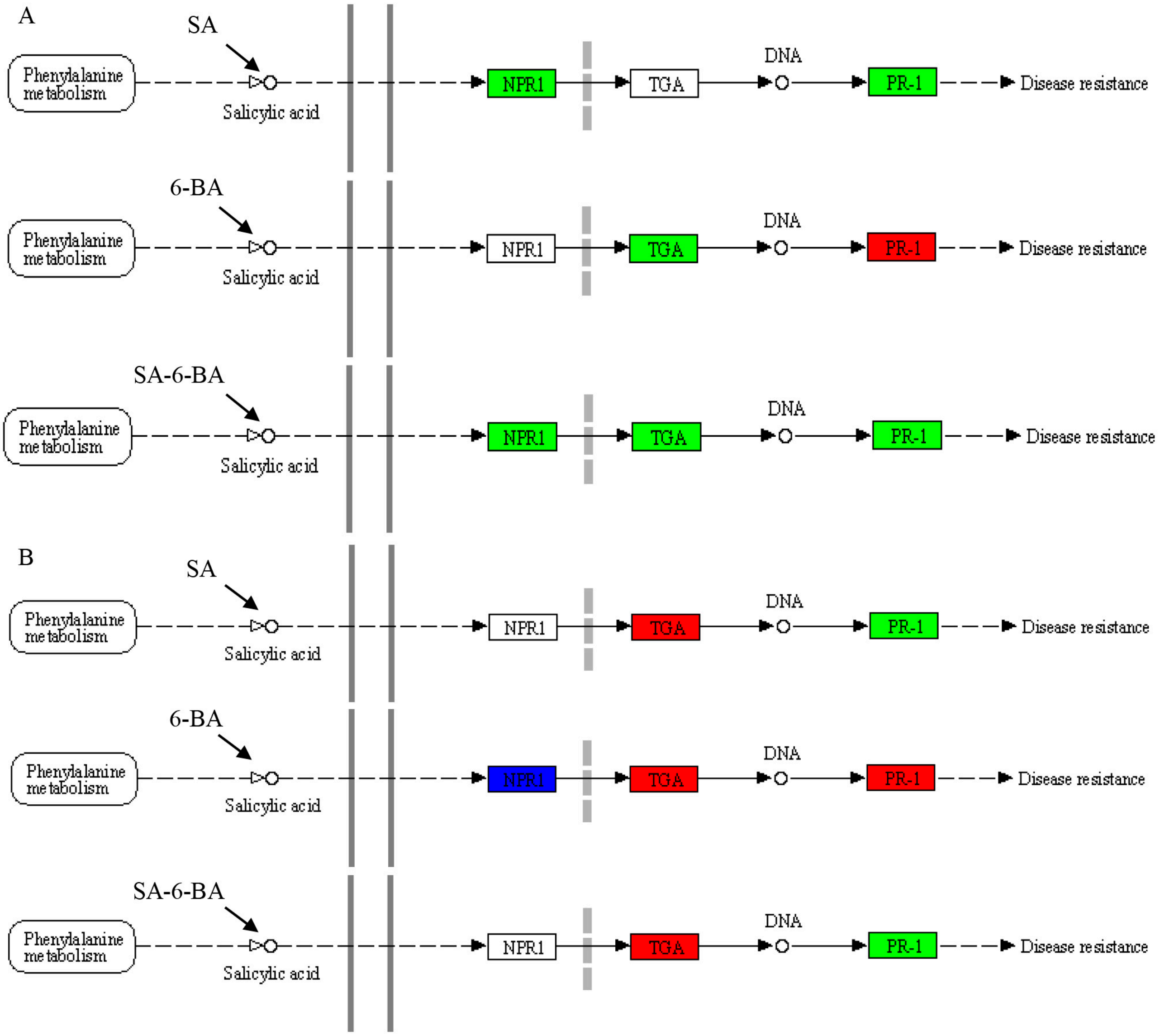

Figure S4. Salicylic acid signaling pathway regulated by exogenous hormones in mesocotyl length.

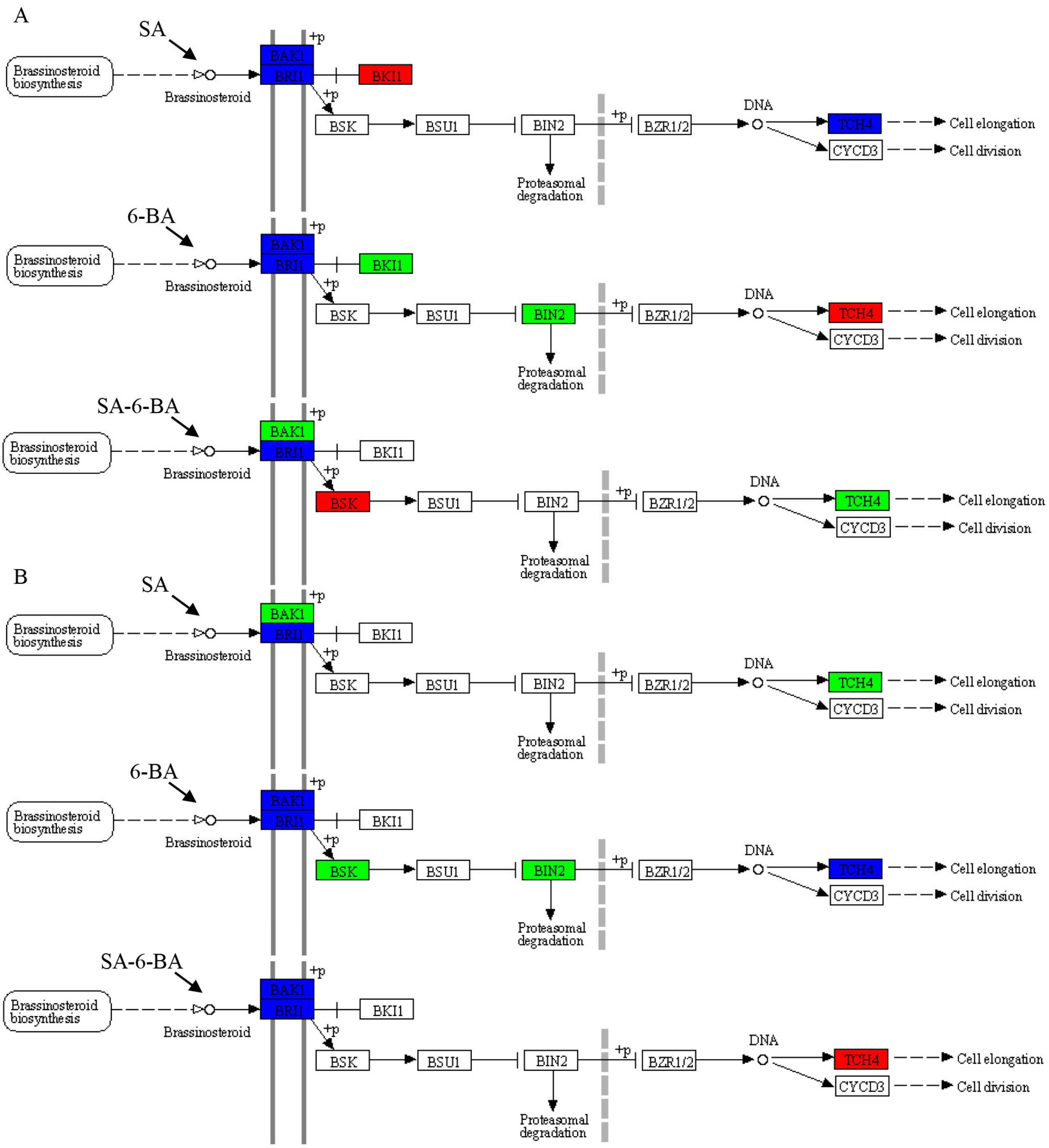

Figure S5. Brassinosteroid signaling pathway regulated by exogenous hormones in mesocotyl length.
